# Factors Associated with Serum Vitamin D Metabolites and Vitamin D Metabolite Ratios in Premenopausal Women

**DOI:** 10.3390/nu13113747

**Published:** 2021-10-23

**Authors:** María José Toribio, Feliciano Priego-Capote, Beatriz Pérez-Gómez, Nerea Fernández de Larrea-Baz, Emma Ruiz-Moreno, Adela Castelló, Pilar Lucas, María Ángeles Sierra, Marina Nieves Pino, Mercedes Martínez-Cortés, María Dolores Luque de Castro, Virginia Lope, Marina Pollán

**Affiliations:** 1 Servicio de Admisión, Hospital General Universitario Gregorio Marañón, 28007 Madrid, Spain; mariajose.toribio@salud.madrid.org; 2 Department of Preventive Medicine, Public Health and Microbiology, Universidad Autónoma de Madrid, 28029 Madrid, Spain; 3 Department of Analytical Chemistry, University of Córdoba, 14014 Córdoba, Spain; q72prcaf@uco.es (F.P.-C.); qa1lucam@uco.es (M.D.L.d.C.); 4 Maimónides Institute of Biomedical Research (IMIBIC), Reina Sofia University Hospital, University of Córdoba, 14004 Córdoba, Spain; 5 Department of Epidemiology of Chronic Diseases, National Center for Epidemiology, Carlos III Institute of Health, 28029 Madrid, Spain; bperez@isciii.es (B.P.-G.); nfernandez@isciii.es (N.F.d.L.-B.); e.ruiz@externos.isciii.es (E.R.-M.); pmlucas@isciii.es (P.L.); masierra@isciii.es (M.Á.S.); mpollan@isciii.es (M.P.); 6 Consortium for Biomedical Research in Epidemiology & Public Health, CIBERESP, 28029 Madrid, Spain; 7 Faculty of Medicine, University of Alcalá, 28871 Alcalá de Henares, Spain; adela.castello@uah.es; 8 Servicio de Prevención y Promoción de la Salud, Madrid Salud, Ayuntamiento de Madrid, 28007 Madrid, Spain; pinoemn@madrid.es (M.N.P.); martinezcme@madrid.es (M.M.-C.)

**Keywords:** Vit D_3_, 25(OH)D_3_, 1,25(OH)_2_D_3_, 24,25(OH)_2_D_3_, vitamin D metabolite ratios

## Abstract

The most representative indicator of vitamin D status in clinical practice is 25(OH)D_3_, but new biomarkers could improve the assessment of vitamin D status and metabolism. The objective of this study is to investigate the association of serum vitamin D metabolites and vitamin D metabolite ratios (VMRs) with potentially influential factors in premenopausal women. This is a cross-sectional study based on 1422 women, aged 39–50, recruited from a Madrid Medical Diagnostic Center. Participants answered an epidemiological and a food frequency questionnaire. Serum vitamin D metabolites were determined using an SPE–LC–MS/MS platform. The association between participant’s characteristics, vitamin D metabolites, and VMRs was quantified by multiple linear regression models. Mean 25(OH)D_3_ concentration was 49.2 + 18.9 nmol/L, with greater deficits among obese, nulliparous, dark-skinned women, and with less sun exposure. A lower R2 ratio (1,25(OH)_2_D_3_/25(OH)D_3_) and a higher R4 (24,25(OH)_2_D_3_/1,25(OH)_2_D_3_) were observed in nulliparous women, with high sun exposure, and those with low caloric intake or high consumption of calcium, vitamin D supplements, or alcohol. Nulliparous women had lower R1 (25(OH)D_3_/Vit D_3_) and R3 (24,25(OH)_2_D_3_/25(OH)D_3_), and older women showed lower R3 and R4. Vitamin D status modified the association of the VMRs with seasons. VMRs can be complementary indicators of vitamin D status and its endogenous metabolism, and reveal the influence of certain individual characteristics on the expression of hydroxylase enzymes.

## 1. Introduction

Vitamin D has been recently hypothesized as a potentially modifiable factor that could reduce the risk of several diseases, such as cardiovascular diseases, diabetes mellitus, multiple sclerosis [1], mental and autoimmune disorders [2], or some types of neoplasms (such as breast cancer) [3]. The US Endocrine Society considers vitamin D sufficiency when serum levels exceed 75 nmol/L [4], and the Institute of Medicine set up a cutoff of 50 nmol/L [5]. According to the last threshold, vitamin D deficiency (<50 nmol/L) has been estimated to affect around 40% of the European [6] and Spanish [7] population.

**Figure 1:**
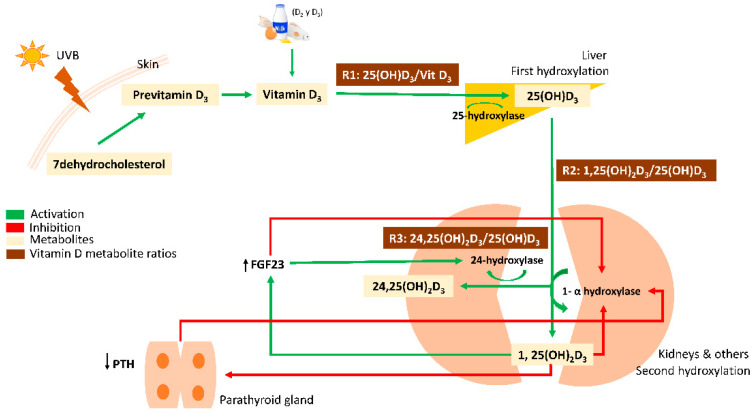
Vitamin D metabolism. Previtamin D_3_ synthesis takes place in the skin by the action of UVB radiation. It undergoes a first hydroxylation in the liver by the enzyme 25-hydroxylase (*CYP2R1*), forming 25(OH)D_3_. Subsequently, 25(OH)D_3_ is hydroxylated to the bioactive 1,25(OH)_2_D_3_ by the enzyme 1α-hydroxylase (*CYP27B1*), predominantly in the kidneys. Vitamin D catabolism is mainly driven by the enzyme 24-hydroxylase (*CYP24A1*), which metabolizes 25(OH)D_3_ to 24,25(OH)_2_D_3._ Vitamin D homeostasis depends on 1,25(OH)_2_D_3_ concentration, which can decrease its own production by directly inhibiting the expression of 1α-hydroxylase or indirectly, by decreasing the synthesis of the parathyroid hormone (↓PTH) or increasing the expression of the phosphaturic factor, fibroblast growth factor 23 (↑FGF23). VMR: Vitamin D Metabolite Ratio.

Vitamin D (calciferol) is mainly produced in the skin by the action of ultraviolet B (UVB) radiation from sunlight, which transforms 7-dehydrocholesterol into previtamin D_3_. This metabolite is considered biologically inactive until it undergoes two enzymatic hydroxylations: the first one in the liver, where previtamin D_3_ is hydroxylated by the 25-hydroxylase (*CYP2R1*) to form 25-hydroxyvitamin D_3_ (25(OH)D_3_), and then in the kidney, where 25(OH)D_3_ is converted to the biologically active hormone calcitriol or 1,25-dihydroxyvitamin D_3_ (1,25(OH)_2_D_3_). This second hydroxylation is mediated by 1α-hydroxylase (*CYP27B1*), which is expressed mainly in the kidney, but also in extra-renal tissues such as breast cells, skin (keratinocytes), immune cells, and bone [4,8]. Vitamin D catabolism takes place in the kidney, where the 24-hydroxylase enzyme (*CYP24A1*) metabolizes 25(OH)D_3_ to 24,25-dihydroxyvitamin D_3_ (24,25(OH)_2_D_3_), the main catabolic metabolite with some biological activity [9]. The crucial control point in vitamin D homeostasis is the renal production of 1,25(OH)_2_D_3_ via 1α-hydroxylase. Calcitriol (1,25(OH)_2_D_3_) can decrease its own production acting directly on the expression of the 1α-hydroxylase or indirectly decreasing parathyroid hormone (PTH) synthesis and, therefore, decreasing 1α-hydroxylase transcription. Rising concentrations of 1,25(OH)_2_D_3_ also increase the expression of the phosphaturic factor, fibroblast growth factor 23 (FGF23), which suppresses the expression of 1α-hydroxylase in the kidney and causes up-regulation of *CYP24A1* expression [9] (Figure 1).

The most abundant circulating vitamin D metabolite is 25(OH)D_3_. Despite not being the biologically active form, it has been the most widely used indicator of vitamin D in most epidemiological studies, due in part to the lack of selective and sensitive methods for the determination of dihydroxymetabolites [10]. Despite its clinical relevance, the determination of vitamin D_3_ metabolites continues to be a challenge, as it provides a more complete snapshot of vitamin D_3_ status due to its physical and chemical properties (hydrophobic nature, thermal and UV instability, and similar structure). In addition, several limitations hinder the utility of 25(OH)D_3_ in clinical practice, such as analytical aspects and interpretation of results [9]. In response to these limitations, new candidate biomarkers have been postulated that could improve the assessment of vitamin D status and metabolism [9]. Among these emerging candidates, vitamin D metabolite ratios (VMRs) are beginning to be used in recent studies [10,11,12,13,14,15,16], since they are not affected by the concentration of vitamin D binding proteins, are good indicators of the expression of hydroxylase enzymes, and could be useful to provide a better assessment of vitamin D status [17].

This study sought to evaluate potentially influential factors in serum levels of vitamin D_3_, 25(OH)D_3_, 1,25(OH)_2_D_3_, 24,25(OH)_2_D_3_, and the four VMRs directly connected by a substrate/product relationship (25(OH)D_3_/VitD_3_, 1,25(OH)_2_D_3_/25(OH)D_3_, 24,25(OH)_2_D_3_/25(OH)D_3_, and 24,25(OH)_2_D_3_/1,25(OH)_2_D_3_) in middle-aged women close to menopause, a period with higher risk of developing vitamin D deficiency [18]. The knowledge of the vitamin D status and its metabolism in this group of women, as well as the sociodemographic factors and lifestyles that are associated, is of great interest to prevent or mitigate bone loss and other conditions related to both menopause and vitamin D deficiency.

## 2. Materials and Methods

### 2.1. Study Population

Between June 2013 and May 2015, 1466 premenopausal women, aged 39 to 50, who worked at the Madrid City Council, were invited to participate in the DDM-Madrid study, aimed to assess the effect of vitamin D on mammographic density. These women were recruited in the Madrid Medical Diagnostic Center (Madrid Salud), where they attended to undergo their routine gynecological check-up. Participants were excluded if they were postmenopausal (at least 1 year without menstruation); were pregnant or breastfeeding; had breast cancer; or had undergone a mastectomy, breast reconstruction, or breast augmentation.

### 2.2. Recruitment and Data Collection

Women were invited to participate in the study by phone, when the selection criteria were verified. Overall participation rate was 88%. The day that each participating woman had her medical examination scheduled, the interviewers administered a standardized epidemiological questionnaire, drew a blood sample, and took anthropometric measurements (height, weight, and waist and hip circumference). The questionnaire collected sociodemographic variables, information on childhood and youth, personal and family medical history, gynecological and obstetric history, work history, skin type and sunbathing habits, sleep habits, tobacco and alcohol consumption, and physical activity. Participants also completed a validated [19] 117-item semi-quantitative food frequency questionnaire that included eating habits during the previous 12 months. Blood samples were centrifuged, aliquoted, and stored at −80 °C in the Carlos III Institute of Health Biobank. The DDM-Madrid study was conducted in accordance with the Declaration of Helsinki guidelines. All participants signed an informed consent, and the protocol was approved by the Ethics and Animal Welfare Committee of the Carlos III Institute of Health. Further details regarding the study design have been previously published [20,21].

### 2.3. Biochemical Analyses

The determination of vitamin D metabolites was carried out in the Metabolomics Unit of the University of Córdoba using an automatic solid-phase extraction unit on-line connected to a liquid chromatograph–tandem mass spectrometer arrangement (SPE-LC-MS/MS). This method was validated by a standard reference material, applying the Vitamin D Standardization Program (VDSP) protocols [22], and according to external quality assurance scheme (DEQAS) [23]. Briefly, 200 µL of filtered serum spiked with deuterated standards of the analytes was introduced for cleanup–chromatographic separation as required tandem mass spectrometry detection. Calibration curves for quantification were obtained using the ratio between the chromatographic peak area of each analyte and that of the corresponding deuterated standard. More information on sample preparation and LC-MS/MS analysis can be found in the article by Mena-Bravo et al. [24], and in Appendix A.

### 2.4. Statistical Methods 

After excluding 27 women whose serum vitamin D levels could not be measured, and 17 women with lack of information in key covariates, the final sample size included 1422 participants. 

Descriptive characteristics of participants were summarized as absolute values and percentages. Geometric means (GM), and the 25th, 50th, and 75th percentiles of Vit D_3_, 25(OH)D_3_, 1,25(OH)_2_D_3_, and 24,25(OH)_2_D_3_, according to women characteristics were also described. Comparisons were also made using the Wald test, with linear regression models adjusted for the weekly sun exposure score, vitamin D intake, and season. The weekly sun exposure score was calculated, taking into account the daily time in sun and the skin area exposed, according to the study by Hanwell et al. [25]. GM of the following VMRs were also calculated: R1: 25(OH)D_3_/Vit D_3_; R2: 1,25(OH)_2_D_3_/25(OH)D_3_; R3: 24,25(OH)_2_D_3_/25(OH)D_3_; and R4: 24,25(OH)_2_D_3_/1,25(OH)_2_D_3_.

Since the distribution of metabolite and VMR concentrations were positively skewed, the values were log-transformed to improve normality. To assess their association with women characteristics, we estimated geometric mean ratios (GMR) and 95% confidence intervals through multiple linear regression models, adjusted for weekly sun exposure score, vitamin D intake and season, and for those variables that were associated with each metabolite’s concentration (*p* < 0.10) in the above-described Wald test analysis. For VMRs, models were adjusted for the same 3 mentioned variables plus those variables that, in this last analysis, showed to be relevant for any of the two metabolites of each ratio (*p* < 0.05). Differences in the associations of VMR according to vitamin D status (deficiency: 25(OH)D_3_ < 50 nmol/L and non-deficiency: 25(OH)D_3_ > 50 nmol/L) were also explored. Possible effect modifications were tested using the likelihood ratio test. Finally, to take into account the problem of multiple comparisons or multiple testing, *p*-values were also suitably adjusted by controlling the expected proportion of false positives, as proposed by Benjamini and Hochberg [26]. All analyses were performed using STATA/MP 14.0 software.

## 3. Results

The mean age of the participants was 44 years. As can be seen in Table 1, 23% of the women were overweight, and almost 10% were obese. Most were university graduates (61%). The percentage of nulliparous, non-smoking, abstemious, and sedentary women was 24%, 39%, 20%, and 42%, respectively. Hypercholesterolemia was reported in 13% of the women, and 10% were in treatment with corticosteroids. Most of the participants had a type IV skin phototype. The mean (+standard deviation) consumption of calories and calcium was 1976 + 681 Kcal/day and 1129 + 491 mg/day, respectively. Sun exposure, according to the weekly sun exposure score, was low in 47% of women, and vitamin D intake was lower than 5 µg/day in 72%. Most of the samples were obtained in spring (33%) and fall (29%).

The mean 25(OH)D_3_ concentration was 49.2 + 18.9 nmol/L. More than half of the participants (59%) had vitamin D deficiency (25(OH)D_3_ < 50 nmol/L). Serum levels were significantly higher in women with adequate body mass index (BMI), with one or two children, with higher sun exposure, in the most physically active women, in those taking vitamin D supplements, and in samples collected during the summer months. Both native vitamin D and 24,25(OH)_2_D_3_ showed the same pattern as 25(OH)D_3_ regarding BMI, physical activity, sun exposure, and season. 24,25(OH)_2_D_3_ levels were also lower in nulliparous and in current smokers, were inversely associated with age, and positively associated with calcium and vitamin D intake. Vitamin D_3_ levels were also higher in corticosteroid users. Finally, 1,25(OH)_2_D_3_ levels were higher in nulliparous women with higher calorie intake and in samples obtained in winter (Table 1). 

Table 2 shows the association between the concentrations of vitamin D metabolites and women’s characteristics. Obese women had lower levels of Vit D_3_, 25(OH)D_3_ and 24,25(OH)_2_D_3_, while physically active women had higher concentrations of these metabolites. Parous women, as well as those taking vitamin D supplements, had higher concentrations of 25(OH)D_3_ and 24,25(OH)_2_D_3_. Sun exposure was positively associated with Vit D_3_, 25(OH)D_3_, and 24,25(OH)_2_D_3_ levels. Concentrations of these three metabolites were also higher in samples obtained in summer, and lower in the samples collected in winter. Women using corticosteroids had higher Vit D_3_ concentrations (GMR = 1.09; 95%CI = 1.01–1.17). Current smokers presented lower levels of 24,25(OH)_2_D_3_ (GMR = 0.93; 95%CI = 0.87–0.99). Phototype V-VI was associated with decreased 25(OH)D_3_ concentrations (GMR = 0.90; 95%CI = 0.83–0.99). Finally, participants with higher calcium intake and lower calorie consumption had lower levels of 1,25(OH)_2_D_3_. 

With respect to VMR, older women presented lower values of R3 (GMR _> 45 years_ = 0.94; 95%CI = 0.90–0.98) and R4 (GMR_>45 years_ = 0.92; 95%CI = 0.88–0.97). Nulliparous women presented lower values of R1 (GMR = 0.92; 95%CI = 0.85–0.99), R3 (GMR = 0.95; 95%CI = 0.91–1.00), and R4 (GMR = 0.84; 95%CI = 0.79–0.90), but higher values of R2 (GMR = 1.13; 95%CI = 1.06–1.19). This last ratio presented an inverse association with alcohol consumption (GMR_>10g/day_ = 0.90; 95%CI = 0.83–0.98). Physically active women had higher R4 values (GMR_>12 MET-h/week_ = 1.09; 95%CI = 1.02–1.16), while current smokers had lower values of this ratio. While R2 was inversely associated with sun exposure (GMR_WSES=29–56_ = 0.82; 95%CI = 0.76–0.89) and calcium intake (GMR_>1246.9 mg/day_ = 0.91; 95%CI = 0.85–0.98), and positively with calorie consumption (GMR_>2144.0 kcal/day_ = 1.13; 95%CI = 1.05–1.22), the association of R4 with these three variables was exactly the opposite. Women who took vitamin D supplements showed higher R1 (GMR = 1.21; 95%CI = 1.08–1.35) and R4 values (GMR = 1.10; 95%CI = 1.01–1.21) and lower R2 values (GMR = 0.86; 95%CI = 0.79–0.93). All ratios showed seasonal variations, with R1 values being higher in fall, R2 values in winter, and R3 and R4 values in summer and fall. In contrast, the lowest levels were obtained in summer for R1, in spring for R2 and R3, and in winter for R4 (Table 3).

Table 4 and Table 5 show the association of VMR with women’s characteristics in participants with deficient (25(OH)D_3_ < 50 nmol/L) and non-deficient (25(OH)D_3_ > 50 nmol/L) serum vitamin D levels. For most of the studied associations, no differences were observed between these two groups. However, among participants with vitamin D deficiency, those who were taking corticosteroids had lower values of the R1 ratio than those who did not take corticosteroids, while no statistically significant differences were observed in participants with non-deficient levels of vitamin D (P-het = 0.027). The association of hypercholesterolemia treated with statins with VMR (decreasing the R2 values and increasing the R4 values) was only observed among women with non-deficient serum vitamin D levels. Finally, vitamin D status modified the association of the first three ratios with the season of the year, while R1 was only associated in women with sufficient levels of vitamin D (P-het < 0.001), R2 was altered only in women with deficient levels of this vitamin (P-het = 0.020), and the high R3 value in summer was only observed among participants with non-deficient vitamin D concentrations.

## 4. Discussion

To our knowledge, this is the first study providing information on the association of serum VMRs with several sociodemographic and lifestyle-related characteristics in premenopausal women. Our results show a notable vitamin D deficiency in the participating women, as well as the influence of certain factors (such as age, parity, and several lifestyles) on the vitamin D serum levels, its metabolites, and VMR.

Vitamin D deficiency (<50 nmol/L of 25(OH)D_3_) is a global problem [4] that affects around 40% of the European population [6,27], and the Southern European countries [7]. In Spain, despite abundant sunshine, it has been estimated that 40% of the Spanish adult population have serum concentrations of 25(OH)D_3_ below 50 nmol/L, and 18% below 25 nmol/L. These figures are 35% and 27% when we refer exclusively to the elderly population and postmenopausal women [7]. In our study, more than half (59%) of the participants had deficient levels of vitamin D, and only 9% had optimal levels (>75 nmol/L). Nulliparous women, and those with obesity or with darker skin, presented lower levels of 25(OH)D_3_, while women with greater sun exposure, those who took vitamin D supplements, were physically active, drank more alcohol, and those whose samples were collected in summer had higher concentrations. Regarding BMI, our results are in line with other Spanish [28] and international studies [29,30], in which obesity was significantly associated with lower 25(OH)D_3_ levels. Circulating vitamin D concentrations are partially determined by genetic factors, and play an important role in the process of adipogenesis and inflammation status in adipocytes and adipose tissue [31]. Due to its fat solubility, vitamin D is retained by the body fat mass, resulting in lower availability of vitamin D for metabolic function in obese people [31,32]. Regarding parity, although a recent study has shown no association [33], Andersen et al. observed that the prevalence of vitamin D insufficiency was less frequent in nulliparous women [34]. The lower levels detected in our nulliparous participants could be due to lifestyles that imply less sun exposure or greater protection from the sun, different eating habits (egg and dairy products consumption was significantly lower in nulliparous participants), or the involvement of endogenous factors (such as the influence of hormones on vitamin D metabolism). Several observational studies have shown that vitamin D deficiency is a risk marker for reduced female fertility and various adverse pregnancy outcomes [35,36]. Leisure-time physical activity appears to be an effective manner of maintaining adequate vitamin D concentrations [37]. Such association has often been attributed to confounding factors, but recent studies indicate that exercise may have a direct and causal effect on vitamin D status, possibly through the mobilization of adipose-derived vitamin D and/or 25(OH)D_3_ [38], or through an increase in muscle use producing the release of 25OHD from its interior [39]. The association between alcohol consumption and vitamin D serum levels remains controversial, although recent studies, with large sample sizes, showed positive associations [40]. Consistent with our findings, other factors related to sun exposure, such as short time spent in the sun, low amount of skin surface exposed, samples collected in winter/early spring, and increased skin pigmentation have been associated with higher risk of 25(OH)D_3_ deficiency in the literature [41]. Finally, and as expected, the intake of vitamin D supplements increased serum levels of 25(OH)D_3_. However, the intake of these supplements is very infrequent, both among the women of our study and in Spain in general [42]. Only 19% of our participants took the 5 µg/day of vitamin D recommended by the Spanish Federation of Societies of Nutrition, Food, and Dietetics (FESNAD) in 2010 [43], and only 0.4% took the 15 µg/day recommended in 2019 by the European Food Safety Authority (EFSA) for the adult population [44].

Although 25(OH)D_3_ is still recommended as the marker of choice by current guidelines from scientific organizations, growing evidence indicates significant limitations that hamper the utility of this analyte in clinical practice, including analytical aspects and interpretation of results [9]. VMRs are promising emerging biomarkers that may provide additional information in assessing vitamin D status [9,45]. The first ratio (25(OH)D_3_/Vit D_3_), represents the activity of 25-hydroxylase enzyme in the liver, which is the main enzyme responsible for the conversion of vitamin D_3_ to the main circulating form of this vitamin, the 25(OH)D_3_. The values of this ratio were similar to those described in the study by Mena-Bravo et al. [10]. This ratio was higher in women taking supplements and among participants with sufficient 25(OH)D_3_ levels whose samples were collected in fall or winter, and lower among nulliparous women and corticosteroid users with deficient vitamin D levels. 

The second ratio (1,25(OH)_2_D_3_/25(OH)D_3_) represents the 1α-hydroxylase activity, an enzyme encoded by the *CYP27B1* gene in the kidney, where 25(OH)D_3_ is converted to the active 1,25(OH)_2_D_3_. We found a higher ratio in obese women (mainly in those with deficient serum vitamin D levels), in nulliparous women, in those with more caloric diets, and in women with deficient vitamin D concentrations whose samples were collected in winter. On the contrary, this ratio was lower in women with higher consumption of alcohol, calcium, vitamin D supplements, and statin users; in women with greater sun exposure; and in samples collected in spring and summer (both results only detected in women with vitamin D deficiency). The values of this ratio in our participants are slightly higher than those described by Mena-Bravo et al. [10] (average ± SD: 0.0029 ± 0.002) and, although we have not found studies reporting characteristics associated with this ratio, there is evidence that high dietary calcium intake reduces 1α-hydroxylase activity (reflected in a lower R2 ratio), while low calcium intake down-regulates 24-hydroxylase expression [9,46].

The third ratio (24,25(OH)_2_D_3_/25(OH)D_3_) is mediated by the *CYP24A1* gene that encodes the enzyme 24-hydroxylase, which catalyzes the conversion of 25(OH)D_3_ into 24,25(OH)_2_D_3_. When sufficient amounts of biologically active vitamin D are available, *CYP24A1* is up-regulated and more 24,25(OH)_2_D_3_ is formed [9]. This ratio may be of potential use as an indicator of vitamin D deficiency and as a predictor of the change in 25(OH)D_3_ after vitamin D supplementation. It may also help explain some of the inter-individual differences in the response of serum 25(OH)D_3_ to the same administered dose of vitamin D [9,13,47,48,49]. In some studies, low levels of this ratio seem to be related to the increasing all-cause mortality in patients with chronic kidney disease and risk of hip fracture in older adults [16,50]. However, in our study, we found no differences in this ratio between the participants that were taking vitamin D supplements and those who did not (regardless their vitamin D status), in line with what was observed in previous studies [14,45], and contrary to what was observed in Tang’s study [11]. Older women, nulliparous women, and those whose samples were collected in spring had a lower R3 ratio, although the association of this ratio with season varied as a function of vitamin D levels. Regarding R3 mean values, two previous studies have described figures that are in line with those obtained in our study [10,11].

Finally, the fourth ratio (24,25(OH)_2_D_3_/1,25(OH)_2_D_3_) could also be a good indicator of vitamin D status. Tang et al., observed an inverse correlation between the 1,25(OH)_2_D_3_/24,25(OH)_2_D_3_ ratio and the 25(OH)D_3_ levels, so that when vitamin D levels were insufficient, the production of 1,25(OH)_2_D_3_ was favored to the detriment of its conversion to 24,25(OH)_2_D_3_ [11]. This phenomenon is also compatible with our results, since the GM of R4 was lower in women with vitamin D deficiency than in those with non-deficient levels. The R4 ratio was higher in women who consumed calcium and vitamin D supplements, in participants with high sun exposure, in those who were physically active and in samples collected in summer and fall. On the contrary, the oldest women, nulliparous women, those whose samples were collected in winter or spring, and the participants with non-deficient levels of vitamin D who consumed many calories had a lower R4 ratio.

The cross-sectional design of this study limits the possibility of establishing a temporal relationship between the exposures and vitamin D metabolite levels. This sample includes only premenopausal women recruited from a single center, so the results cannot be extrapolated to the general population. In addition, only a single blood sample was collected at the beginning of the study, so the participants’ usual vitamin D status may not have been adequately reflected. On the other hand, given that we have used a novel approach to provide a more complete picture of vitamin D3 metabolism, the results of this study should be considered as hypothesis-generating and should be viewed with caution. Precisely, due to its hypothesis-generating approach and the exploratory nature of the study, corrections for multiple testing were not applied in the main analyses [51], although results adjusted using the Benjamini and Hochberg method [26] are reported in the Supplementary Material (Tables S1 and S2). Finally, even though we have included the main variables described in the literature as associated with vitamin D levels in our models, the possibility of residual confounding cannot be ruled out.

The greatest strength of our study is its novelty. In a relatively large sample of participants, we were able to quantify vitamin D metabolites, the ratios between them, and the factors that contribute to explain metabolic variations. In addition, SPE-LC-MS/MS is a sensitive automated method for the analysis of serum vitamin D and metabolites that provides reliable and robust results. This method was validated by a standard reference material and according to DEQAS [23].

## 5. Conclusions

In general, vitamin D metabolite profile in nulliparous women and older women was compatible with lower activity of the enzyme 24-hydroxylase, which catabolizes 25(OH)_2_D_3_ to 24,25(OH)_2_D_3_. Furthermore, nulliparous women and those who consumed more calories showed an increase in calcitriol levels to the detriment of the concentrations of the other two metabolites. The opposite was observed among the participants with greater consumption of calcium, alcohol, or with greater sun exposure. Finally, the association of VMR with seasons was different depending on vitamin D status. These results highlight the added value of VMR as complementary indicators of vitamin D status and its endogenous metabolism, being considered better predictors of vitamin D treatment response and clinically important outcomes. The results also reveal the potential contribution of certain factors in the greater or lesser expression/activity of hydroxylase enzymes.

## Figures and Tables

**Table 1:** Characteristics of the participants and distribution of vitamin D metabolite levels according to them.

			Vit D_3 (_nmol/L)				25(OH)D_3_ (nmol/L)				1,25(OH)_2_D_3_ (pmol/L)				24,25(OH)_2_D_3_ (nmol/L)			
Characteristics	*n*	(%)	P25	P50	P75	*p*-Val ^a^	P25	P50	P75	*p*-Val ^a^	P25	P50	P75	*p*-Val ^a^	P25	P50	P75	*p*-Val ^a^
Total	1422	(100)	4.5	6.4	8.5		35.9	45.8	58.7		96.9	111.1	125.7		2.3	3.1	4.0	
Age																		
<45	758	(53.3)	4.3	6.2	8.4	0.827	35.5	45.9	58.8	0.497	96.3	110.9	126.1	0.450	2.4	3.2	4.1	0.002
≥45	664	(46.7)	4.6	6.5	8.6		36.2	45.7	58.4		97.5	111.4	125.2		2.1	2.9	4.0	
Educational level																		
Primary school or less	63	(4.4)	4.5	6.4	8.3	0.906	32.5	43.3	55.1	0.813	98.8	112.3	128.9	0.569	2.2	2.9	4.0	0.798
Secondary school	487	(34.3)	4.5	6.4	8.5		36.8	48.0	58.8		97.0	111.7	126.1		2.3	3.1	4.1	
University graduate	871	(61.3)	4.4	6.4	8.6		35.6	45.1	58.8		96.7	110.8	125.4		2.2	3.1	4.0	
Body mass index (kg/m^2^)																		
<20	148	(10.4)	4.4	6.4	9.8	<0.001	35.6	44.8	60.4	0.010	93.1	109.5	125.2	0.532	2.1	3.0	4.1	0.006
20–25	810	(57)	4.6	6.5	8.8		36.9	46.9	58.5		97.1	111.0	125.7		2.3	3.2	4.1	
25–29	326	(22.9)	4.4	6.2	8.1		35.3	44.7	59.0		97.6	112.5	126.1		2.2	2.9	4.0	
≥ 30	137	(9.6)	4.0	5.4	7.6		34.5	45.3	58.5		97.5	112.2	124.0		2.1	2.8	3.6	
Number of children																		
None	336	(23.6)	4.3	6.3	8.6	0.824	34.4	43.5	55.5	0.004	97.9	113.5	126.5	0.055	2.0	2.8	3.8	<0.001
1	328	(23.1)	4.4	6.2	8.5		36.7	47.5	59.6		96.7	112.5	126.1		2.2	3.2	4.1	
2	677	(47.6)	4.5	6.5	8.5		36.8	47.3	59.2		96.7	109.3	125.0		2.3	3.2	4.1	
>2	81	(5.7)	4.5	6.4	8.9		36.2	44.5	54.8		96.6	110.8	122.3		2.3	3.0	4.2	
Tobacco consumption																		
No	550	(38.7)	4.5	6.3	8.5	0.684	36.8	46.7	59.0	0.164	97.0	111.8	126.2	0.333	2.3	3.1	4.1	0.024
Exsmoker	493	(34.7)	4.5	6.5	8.6		35.8	45.6	59.5		96.7	110.8	125.9		2.3	3.2	4.1	
Current smoker	379	(26.7)	4.4	6.3	8.4		34.5	45.0	56.6		97.1	110.8	124.9		2.1	2.9	3.9	
Alcohol consumption																		
No	249	(19.9)	4.4	6.3	8.6	0.849	34.6	43.7	55.5	0.116	97.5	111.5	125.3	0.823	2.3	3.1	3.9	0.952
<10 g/day	827	(66.1)	4.5	6.4	8.4		36.2	46.4	58.7		96.3	109.5	123.0		2.3	3.0	4.0	
≥10 g/day	176	(14.1)	4.4	6.3	8.5		38.6	47.2	58.3		97.2	112.9	129.7		2.2	3.1	4.2	
Physical activity (MET-h/week)																		
None	592	(41.8)	4.3	6.1	8.3	0.001	34.8	44.0	56.3	0.077	96.8	110.8	126.7	0.309	2.1	2.9	3.9	0.031
≤12	351	(24.8)	4.4	6.2	8.3		36.8	45.6	58.3		96.7	111.6	125.1		2.2	3.1	4.1	
>12	473	(33.4)	4.7	6.7	8.9		38.3	49.3	60.9		96.9	111.0	124.8		2.4	3.2	4.2	
Hypercholesterolemia																		
No	1228	(87.3)	4.4	6.3	8.5	0.117	36.1	46.0	58.6	0.672	97.0	111.4	125.7	0.178	2.3	3.1	4.0	0.955
Yes, not treated	147	(10.4)	5.0	6.5	8.3		35.5	43.9	57.8		98.6	111.4	129.0		2.1	2.9	3.9	
Treated with statins	32	(2.3)	5.6	6.7	9.0		33.5	51.5	81.0		87.3	108.6	123.0		2.4	3.1	5.0	
Ever used corticosteroids																		
No	1272	(89.6)	4.4	6.3	8.5	0.026	35.9	45.8	58.6	0.225	96.9	111.0	125.1	0.299	2.3	3.1	4.0	0.224
Yes	148	(10.4)	5.2	6.7	8.8		37.2	46.4	62.2		98.0	111.8	128.7		2.3	3.2	4.1	
Phototype																		
I-II	117	(8.3)	4.5	6.6	9.1	0.835	38.0	47.1	62.1	0.145	98.3	113.4	129.7	0.652	2.4	3.1	4.1	0.462
III	263	(18.7)	4.3	6.0	8.1		35.5	45.2	58.5		94.6	109.9	123.1		2.2	2.9	3.9	
IV	758	(53.8)	4.5	6.3	8.5		36.3	47.5	59.0		96.8	110.9	125.3		2.3	3.1	4.1	
V-VI	271	(19.2)	4.6	6.5	8.7		33.7	44.4	54.9		99.0	111.8	127.6		2.2	3.1	4.0	
Weekly Sun Exposure Score ^b^																		
<15	534	(47.4)	4.2	6.2	8.2	0.028	33.0	42.2	52.3	<0.001	98.9	113.2	127.6	0.510	2.0	2.8	3.7	0.019
15–28	337	(29.9)	4.4	6.0	8.1		39.3	49.8	64.2		94.1	109.1	124.2		2.5	3.2	4.2	
29–56	255	(22.6)	5.8	7.5	9.5		43.0	54.4	65.6		91.6	104.3	125.3		2.6	3.6	4.6	
Total energy intake (kcal/day) ^c^																		
<1669.2	418	(33.4)	4.3	6.2	8.2	0.896	36.2	45.6	60.7	0.318	95.2	107.3	122.4	0.059	2.2	3.1	4.1	0.728
1669.2–2144.1	417	(33.3)	4.6	6.6	8.6		36.6	47.1	58.7		95.7	111.0	124.9		2.2	3.0	3.9	
>2144.1	417	(33.3)	4.5	6.3	8.5		35.8	45.6	55.8		99.6	112.0	126.6		2.3	3.1	4.0	
Total calcium intake (mg/day) ^c^																		
<892.5	418	(33.4)	4.4	6.3	8.3	0.559	36.5	44.9	57.4	0.413	96.2	111.1	124.9	0.349	2.2	3.0	3.9	0.046
892.5–1246.9	417	(33.3)	4.4	6.5	8.7		35.5	47.4	59.0		97.0	112.0	125.3		2.2	3.1	4.1	
>1246.9	417	(33.3)	4.5	6.4	8.4		36.2	46.0	58.2		97.3	108.5	124.1		2.4	3.1	4.0	
Total vitamin D intake (µg/day) ^d^																		
<5	906	(72.4)	4.5	6.3	8.4	0.945	35.6	45.6	57.0	0.001	96.1	110.8	124.7	0.435	2.2	3.0	3.9	0.003
≥5	237	(18.9)	4.6	6.7	8.8		36.5	45.8	58.1		99.4	111.0	125.7		2.4	3.2	4.1	
Supplements intake	109	(8.7)	4.2	5.7	7.7		38.5	52.8	69.6		97.1	108.4	123.4		2.4	3.2	4.4	
Season																		
Spring	467	(32.8)	5.1	6.6	8.5	<0.001	35.6	45.7	58.1	0.005	93.2	109.9	127.7	<0.001	1.9	2.7	3.6	<0.001
Summer	227	(16)	5.9	8.2	9.9		43.0	55.1	66.4		92.3	105.7	123.8		2.8	3.8	4.8	
Fall	415	(29.2)	4.2	5.5	7.1		37.2	46.9	58.5		97.0	110.6	125.0		2.7	3.5	4.6	
Winter	313	(22)	4.1	5.0	8.6		32.7	40.1	50.7		103.1	115.4	126.7		2.0	2.7	3.3	

P25: percentile 25; P50: median; P75: percentile 75; MET: metabolic equivalent. ^a^ *p*-value adjusted for weekly sun exposure score, vitamin D intake and season. ^b^ Taking into account daily time in sun and skin exposure according to Hanwell et al. ^c^ In tertiles. ^d^ Cut-off point established according to the dietary reference intake for Spanish women aged 40–49 years (*Spanish Federation* of Societies of Nutrition, Food and Dietetics (FESNAD), Ingestas dietéticas de referencia (IDR) para la población española, Eunsa, 2010).

**Table 2:** Association between vitamin D metabolite concentrations and characteristics of women.

	Vit D_3_				25(OH)D_3_				1,25(OH)_2_D_3_				24,25(OH)_2_D_3_			
Characteristics	GM	GMR ^a^	95%CI	*p*-Val	GM	GMR ^b^	95%CI	*p*-Val	GM	GMR ^c^	95%CI	*p*-Val	GM	GMR ^d^	95%CI	*p*-Val
Age																
<45	6.0	1.00			45.9	1.00			108.8	1.00			3.1	1.00		
≥45	6.3	1.02	(0.97–1.06)	0.525	45.9	0.99	(0.95–1.03)	0.650	109.1	1.01	(0.99–1.04)	0.410	2.9	0.93	(0.88–0.98)	0.005
Educational level ^e^																
Primary school or less	5.9	0.99	(0.92–1.07)	0.820	43.3	0.97	(0.90–1.04)	0.359	107.6	0.99	(0.95–1.03)	0.630	2.8	0.99	(0.91–1.08)	0.793
Secondary school	6.1	1.01	(0.97–1.06)	0.599	46.9	1.02	(0.98–1.07)	0.269	109.2	1.00	(0.98–1.03)	0.783	3.0	1.02	(0.97–1.07)	0.506
University graduate	6.1	1.00	(0.95–1.04)	0.872	45.6	1.01	(0.97–1.05)	0.677	108.9	1.01	(0.98–1.03)	0.589	3.0	0.99	(0.95–1.04)	0.807
Body mass index (kg/m^2^)																
<20	6.5	1.00			45.6	1.00			107.5	1.00			2.8	1.00		
20–25	6.3	0.98	(0.90–1.06)	0.566	46.5	1.00	(0.93–1.08)	0.897	109.2	1.00	(0.95–1.04)	0.887	3.1	1.04	(0.95–1.14)	0.359
25–29	5.8	0.92	(0.85–1.01)	0.080	45.5	0.97	(0.89–1.05)	0.404	108.3	0.98	(0.93–1.03)	0.444	2.9	0.99	(0.90–1.09)	0.810
≥30	5.3	0.83	(0.74–0.93)	0.001	43.6	0.90	(0.81–1.00)	0.045	110.4	1.00	(0.94–1.06)	0.887	2.7	0.90	(0.80–1.01)	0.080
Parity																
Parous	6.1	1.00			46.7	1.00			108.6	1.00			3.1	1.00		
nuliparous	6.1	0.98	(0.93–1.04)	0.476	43.4	0.90	(0.86–0.95)	<0.001	110.1	1.02	(0.99–1.05)	0.135	2.7	0.87	(0.81–0.92)	<0.001
Tobacco consumption																
No	6.2	1.00			46.6	1.00			108.5	1.00			3.0	1.00		
Exsmoker	6.2	1.01	(0.96–1.07)	0.642	46.2	1.00	(0.95–1.05)	0.949	109.5	1.01	(0.98–1.04)	0.417	3.0	1.02	(0.96–1.08)	0.589
Current smoker	6.0	0.99	(0.94–1.05)	0.848	44.5	0.96	(0.91–1.01)	0.144	108.8	1.02	(0.98–1.05)	0.326	2.8	0.93	(0.87–0.99)	0.019
Alcohol consumption																
No	6.0	1.00			43.9	1.00			109.1	1.00			3.0	1.00		
<10 g/day	6.2	1.00	(0.94–1.06)	0.973	46.2	1.04	(0.98–1.10)	0.161	107.8	0.98	(0.95–1.02)	0.313	3.0	1.01	(0.94–1.08)	0.794
≥10 g/day	6.1	0.98	(0.90–1.06)	0.533	46.8	1.07	(0.99–1.15)	0.080	110.0	1.00	(0.96–1.05)	0.938	3.0	1.04	(0.95–1.13)	0.441
Physical activity (MET-h/week)																
None	5.9	1.00			44.2	1.00			108.9	1.00			2.8	1.00		
≤12	6.1	1.02	(0.96–1.08)	0.466	45.8	1.04	(0.99–1.10)	0.139	108.2	1.00	(0.97–1.03)	0.952	3.0	1.08	(1.01–1.15)	0.020
>12	6.5	1.08	(1.02–1.14)	0.006	48.2	1.05	(1.00–1.11)	0.067	109.4	1.01	(0.98–1.05)	0.350	3.2	1.08	(1.01–1.15)	0.018
Hypercholesterolemia																
No	6.1	1.00			45.9	1.00			109.0	1.00			3.0	1.00		
Yes, not treated	6.5	1.05	(0.98–1.13)	0.190	45.1	1.02	(0.95–1.10)	0.564	110.3	0.99	(0.95–1.04)	0.790	2.9	1.01	(0.93–1.10)	0.752
Treated with statins	7.0	1.08	(0.93–1.26)	0.298	51.6	1.04	(0.91–1.20)	0.536	103.3	0.93	(0.85–1.01)	0.078	3.2	1.03	(0.88–1.22)	0.691
Ever used corticosteroids																
No	6.1	1.00			45.7	1.00			108.8	1.00			3.0	1.00		
Yes	6.6	1.09	(1.01–1.17)	0.023	47.7	1.05	(0.98–1.12)	0.194	110.5	1.02	(0.98–1.06)	0.301	3.0	1.05	(0.96–1.13)	0.274
Phototype																
I-II	6.3	1.00			47.4	1.00			111.8	1.00			3.1	1.00		
III	5.9	0.93	(0.85–1.03)	0.156	45.7	0.96	(0.87–1.04)	0.310	107.6	0.94	(0.89–0.99)	0.026	2.9	0.97	(0.87–1.08)	0.569
IV	6.1	0.95	(0.87–1.03)	0.209	46.4	0.97	(0.90–1.05)	0.506	108.8	0.97	(0.92–1.01)	0.173	3.0	1.00	(0.91–1.10)	0.980
V-VI	6.3	0.97	(0.88–1.06)	0.474	43.8	0.90	(0.83–0.99)	0.026	110.1	0.98	(0.93–1.04)	0.512	2.9	0.92	(0.83–1.03)	0.146
Weekly sun exposure score ^f^																
<15	5.9	1.00			41.9	1.00			110.7	1.00			2.7	1.00		
15–28	6.0	0.96	(0.91–1.02)	0.202	49.8	1.13	(1.07–1.20)	<0.001	107.2	0.98	(0.94–1.03)	0.447	3.2	1.11	(1.05–1.19)	0.001
29–56	7.4	1.08	(1.00–1.16)	0.044	53.1	1.16	(1.08–1.24)	<0.001	105.8	0.95	(0.87–1.04)	0.267	3.4	1.11	(1.03–1.21)	0.010
Total energy intake (kcal/day) ^g^																
<1669.2	6.0	1.00			46.1	1.00			106.7	1.00			3.0	1.00		
1669.2–2144.0	6.3	1.04	(0.98–1.10)	0.222	46.6	1.00	(0.95–1.05)	0.947	107.8	1.01	(0.97–1.04)	0.698	2.9	0.97	(0.91–1.04)	0.423
>2144.0	6.2	1.00	(0.94–1.06)	0.944	44.8	0.97	(0.91–1.02)	0.228	110.6	1.04	(1.00–1.07)	0.042	3.0	0.97	(0.90–1.04)	0.387
Total calcium intake (mg/day) ^g^																
<892.5	6.0	1.00			45.2	1.00			108.4	1.00			2.9	1.00		
892.5–1246.9	6.2	1.03	(0.97–1.09)	0.382	46.1	1.01	(0.96–1.07)	0.711	109.3	1.00	(0.97–1.03)	0.930	3.0	1.03	(0.97–1.10)	0.362
>1246.9	6.2	0.99	(0.94–1.06)	0.863	46.2	1.02	(0.97–1.08)	0.442	107.5	0.96	(0.92–1.00)	0.041	3.1	1.06	(0.99–1.13)	0.103
Total vitamin D intake (µg/day) ^h^																
<5	6.1	1.00			45.1	1.00			108.0	1.00			2.9	1.00		
≥5	6.4	1.04	(0.98–1.10)	0.219	45.7	1.01	(0.95–1.06)	0.807	110.0	1.01	(0.98–1.05)	0.487	3.1	1.06	(0.99–1.13)	0.102
Supplements intake	5.8	0.96	(0.88–1.04)	0.317	52.8	1.18	(1.09–1.27)	<0.001	107.7	1.00	(0.96–1.05)	0.994	3.2	1.12	(1.02–1.22)	0.016
Season ^e^																
Spring	6.4	1.05	(1.01–1.09)	0.013	46.2	1.02	(0.99–1.06)	0.195	107.0	0.97	(0.95–0.99)	0.003	2.6	0.88	(0.84–0.91)	<0.001
Summer	7.5	1.13	(1.06–1.19)	<0.001	53.6	1.06	(1.01–1.12)	0.021	107.3	0.99	(0.96–1.02)	0.452	3.6	1.12	(1.05–1.19)	<0.001
Fall	5.5	0.92	(0.89–0.96)	<0.001	46.0	0.99	(0.96–1.03)	0.789	108.2	1.00	(0.97–1.02)	0.685	3.4	1.14	(1.09–1.19)	<0.001
Winter	5.6	0.92	(0.88–0.96)	0.001	40.6	0.92	(0.88–0.97)	0.001	114.1	1.05	(1.02–1.08)	0.001	2.6	0.89	(0.85–0.94)	<0.001

GM: geometric mean; MET: metabolic equivalent. ^a^ Geometric mean ratio adjusted for body mass index, physical activity, use of corticosteroids, weekly sun exposure score, vitamin D intake, and season. ^b^ Geometric mean ratio adjusted for body mass index, parity, physical activity, weekly sun exposure score, vitamin D intake, and season. ^c^ Geometric mean ratio adjusted for parity, energy intake, weekly sun exposure score, vitamin D intake, and season. ^d^ Geometric mean ratio adjusted for age, body mass index, parity, tobacco, physical activity, weekly sun exposure score, calcium intake, vitamin D intake, and season. ^e^ Using the geometric mean as the reference. ^f^ Taking into account daily time in sun and skin exposure according to Hanwell et al. ^g^ In tertiles. ^h^ Cut-off point established according to the dietary reference intake for Spanish women aged 40–49 years (*Spanish Federation* of Societies of Nutrition, Food and Dietetics (FESNAD), Ingestas dietéticas de referencia (IDR) para la población española, Eunsa, 2010).

**Table 3:** Association between vitamin D metabolite ratios and characteristics of women.

	R1: 25(OH)D_3_ /Vit D_3_				R2: 1,25(OH)_2_D_3_ /25(OH)D_3_				R3: 24,25(OH)_2_D_3_/25(OH)D_3_				R4: 24,25(OH)_2_D_3_/1,25(OH)_2_D_3_			
Characteristics	GM	GMR ^a^	95%CI	*p*-Val	GM	GMR ^b^	95%CI	*p*-Val	GM	GMR ^c^	95%CI	*p*-Val	GM	GMR ^d^	95%CI	*p*-Val
Age																
<45	7.66	1.00			2.4 × 10^−3^	1.00			0.07	1.00			28.24	1.00		
≥45	7.31	0.98	(0.92–1.04)	0.509	2.4 × 10^−3^	1.02	(0.97–1.07)	0.498	0.06	0.94	(0.90–0.98)	0.004	26.13	0.92	(0.88–0.97)	0.003
Educational level ^e^																
Primary school or less	7.29	0.99	(0.89–1.10)	0.815	2.5 × 10^−3^	1.00	(0.92–1.08)	0.978	0.07	1.02	(0.95–1.09)	0.528	26.48	1.01	(0.93–1.10)	0.786
Secondary school	7.65	1.00	(0.94–1.07)	0.907	2.3 × 10^−3^	0.99	(0.94–1.04)	0.676	0.06	0.99	(0.95–1.03)	0.597	27.42	1.00	(0.95–1.06)	0.864
University graduate	7.43	1.01	(0.95–1.07)	0.781	2.4 × 10^−3^	1.01	(0.96–1.06)	0.632	0.06	0.99	(0.95–1.03)	0.602	27.19	0.98	(0.93–1.04)	0.523
Body mass index (kg/m^2^)																
<20	7.04	1.00			2.4 × 10^−3^	1.00			0.06	1.00			25.97	1.00		
20–25	7.35	1.04	(0.93–1.16)	0.504	2.3 × 10^−3^	0.99	(0.91–1.08)	0.846	0.07	1.05	(0.97–1.12)	0.216	28.13	1.06	(0.97–1.16)	0.199
25–29	7.80	1.06	(0.94–1.20)	0.319	2.4 × 10^−3^	1.03	(0.94–1.13)	0.565	0.06	1.04	(0.96–1.13)	0.355	26.86	1.02	(0.92–1.13)	0.655
≥30	8.15	1.09	(0.94–1.27)	0.234	2.5 × 10^−3^	1.11	(0.99–1.25)	0.067	0.06	1.01	(0.91–1.11)	0.887	24.35	0.91	(0.80–1.03)	0.151
Parity																
Parous	7.60	1.00			2.3 × 10^−3^	1.00			0.07	1.00			28.13	1.00		
nuliparous	7.16	0.92	(0.85–0.99)	0.030	2.5 × 10^−3^	1.13	(1.06–1.19)	<0.001	0.06	0.95	(0.91–1.00)	0.046	24.54	0.84	(0.79–0.90)	<0.001
Tobacco consumption																
No	7.56	1.00			2.3 × 10^−3^	1.00			0.07	1.00			27.94	1.00		
Exsmoker	7.50	1.00	(0.93–1.07)	0.908	2.4 × 10^−3^	1.01	(0.95–1.07)	0.719	0.07	1.01	(0.97–1.06)	0.580	27.53	1.00	(0.94–1.06)	0.974
Current smoker	7.38	0.99	(0.91–1.07)	0.783	2.4 × 10^−3^	1.04	(0.98–1.11)	0.208	0.06	0.96	(0.91–1.02)	0.163	25.88	0.93	(0.87–0.99)	0.028
Alcohol consumption																
No	7.26	1.00			2.5 × 10^−3^	1.00			0.07	1.00			27.08	1.00		
<10 g/day	7.50	1.05	(0.97–1.14)	0.248	2.3 × 10^−3^	0.93	(0.88–0.99)	0.026	0.06	0.97	(0.92–1.03)	0.301	27.52	1.05	(0.98–1.13)	0.134
≥10 g/day	7.70	1.10	(0.99–1.23)	0.076	2.4 × 10^−3^	0.90	(0.83–0.98)	0.018	0.06	0.97	(0.90–1.04)	0.358	26.84	1.08	(0.98–1.18)	0.120
Physical activity (MET-h/week)																
None	7.49	1.00			2.5 × 10^−3^	1.00			0.06	1.00			25.92	1.00		
≤12	7.58	1.03	(0.95–1.11)	0.539	2.4 × 10^−3^	0.95	(0.90–1.01)	0.133	0.06	1.03	(0.98–1.09)	0.222	27.52	1.10	(1.03–1.17)	0.006
>12	7.44	0.97	(0.90–1.05)	0.476	2.3 × 10^−3^	0.96	(0.90–1.02)	0.156	0.07	1.03	(0.98–1.08)	0.237	28.83	1.09	(1.02–1.16)	0.008
Hypercholesterolemia																
No	7.56	1.00			2.0 × 10^−3^	1.00			0.06	1.00			27.22	1.00		
Yes, not treated	6.96	0.96	(0.86–1.06)	0.414	2.4 × 10^−3^	0.98	(0.90–1.06)	0.596	0.06	0.99	(0.93–1.06)	0.801	25.96	1.02	(0.93–1.11)	0.708
Treated with statins	7.38	0.97	(0.80–1.19)	0.794	2.0 × 10^−3^	0.88	(0.75–1.03)	0.119	0.06	0.99	(0.87–1.14)	0.916	31.14	1.12	(0.94–1.33)	0.200
Ever used corticosteroids																
No	7.53	1.00			2.4 × 10^−3^	1.00			0.06	1.00			27.24	1.00		
Yes	7.21	0.96	(0.87–1.06)	0.408	2.3 × 10^−3^	0.98	(0.91–1.06)	0.701	0.06	1.01	(0.94–1.08)	0.823	27.36	1.02	(0.94–1.11)	0.610
Phototype																
I-II	7.46	1.00			2.4 × 10^−3^	1.00			0.06	1.00			27.33	1.00		
III	7.73	1.02	(0.89–1.16)	0.777	2.4 × 10^−3^	0.99	(0.89–1.09)	0.799	0.06	1.02	(0.94–1.11)	0.659	26.93	1.04	(0.93–1.16)	0.509
IV	7.60	1.02	(0.91–1.15)	0.693	2.3 × 10^−3^	1.00	(0.91–1.09)	0.966	0.06	1.03	(0.96–1.11)	0.429	27.43	1.04	(0.94–1.15)	0.412
V-VI	6.99	0.93	(0.82–1.06.)	0.296	2.5 × 10^−3^	1.09	(0.98–1.20)	0.106	0.07	1.02	(0.94–1.12)	0.596	26.50	0.95	(0.85–1.06)	0.381
Weekly sun exposure score ^f^																
<15	7.14	1.00			2.6 × 10^−3^	1.00			0.06	1.00			24.42	1.00		
15–28	8.33	1.16	(1.08–1.26)	<0.001	2.2 × 10^−3^	0.88	(0.83–0.93)	<0.001	0.06	0.98	(0.93–1.04)	0.560	29.46	1.12	(1.04–1.19)	0.001
29–56	7.19	1.08	(0.97–1.19)	0.153	2.0 × 10^−3^	0.82	(0.76–0.89)	<0.001	0.06	0.96	(0.90–1.03)	0.241	32.16	1.15	(1.05–1.25)	0.002
Total energy intake (kcal/day) ^g^																
<1669.2	7.75	1.00			2.3 × 10^−3^	1.00			0.06	1.00			27.82	1.00		
1669.2–2144.0	7.41	0.96	(0.89–1.04)	0.277	2.3 × 10^−3^	1.04	(0.97–1.10)	0.258	0.06	0.99	(0.95–1.05)	0.847	27.24	0.96	(0.89–1.02)	0.196
>2144.0	7.28	0.97	(0.89–1.05)	0.430	2.5 × 10^−3^	1.13	(1.05–1.22)	0.001	0.07	1.04	(0.98–1.09)	0.179	26.95	0.93	(0.86–1.00)	0.066
Total calcium intake (mg/day) ^g^																
<892.5	7.54	1.00			2.4 × 10^−3^	1.00			0.06	1.00			26.30	1.00		
892.5–1246.9	7.39	0.98	(0.91–1.06)	0.633	2.4 × 10^−3^	0.99	(0.93–1.05)	0.748	0.06	1.02	(0.97–1.08)	0.401	27.26	1.03	(0.96–1.10)	0.446
>1246.9	7.50	1.03	(0.95–1.12)	0.453	2.3 × 10^−3^	0.91	(0.85–0.98)	0.015	0.07	1.03	(0.98–1.09)	0.211	28.49	1.12	(1.03–1.21)	0.005
Total vitamin D intake (µg/day) ^h^																
<5	7.39	1.00			2.4 × 10^−3^	1.00			0.06	1.00			26.81	1.00		
≥5	7.13	0.97	(0.89–1.05)	0.384	2.4 × 10^−3^	1.01	(0.95–1.08)	0.714	0.07	1.06	(1.01–1.12)	0.023	28.28	1.03	(0.96–1.11)	0.377
Supplements intake	9.11	1.21	(1.08–1.35)	0.001	2.0 × 10^−3^	0.86	(0.79–0.93)	<0.001	0.06	0.96	(0.89–1.03)	0.226	29.77	1.10	(1.01–1.21)	0.038
Season ^e^																
Spring	7.17	0.98	(0.94–1.04)	0.546	2.3 × 10^−3^	0.94	(0.91–0.98)	0.003	0.06	0.86	(0.83–0.88)	<0.001	24.22	0.91	(0.87–0.95)	<0.001
Summer	7.15	0.93	(0.86–1.01)	0.078	2.0 × 10^−3^	0.95	(0.90–1.01)	0.129	0.07	1.05	(1.00–1.11)	0.043	33.53	1.12	(1.05–1.20)	0.001
Fall	8.29	1.08	(1.02–1.14)	0.005	2.4 × 10^−3^	0.99	(0.95–1.03)	0.701	0.07	1.14	(1.10–1.19)	<0.001	31.71	1.15	(1.10–1.20)	<0.001
Winter	7.23	1.01	(0.94–1.08)	0.820	2.8 × 10^−3^	1.12	(1.06–1.18)	<0.001	0.06	0.97	(0.93–1.01)	0.149	22.81	0.86	(0.81–0.91)	<0.001

GM: geometric mean; MET: metabolic equivalent. ^a^ Geometric mean ratio adjusted for body mass index, parity, physical activity, use of corticosteroids, phototype, weekly sun exposure score, vitamin D intake, and season. ^b^ Geometric mean ratio adjusted for body mass index, parity, phototype, weekly sun exposure score, energy intake, calcium intake, vitamin D intake, and season. ^c^ Geometric mean ratio adjusted for age, body mass index, parity, tobacco, physical activity, phototype, weekly sun exposure score, vitamin D intake, and season. ^d^ Geometric mean ratio adjusted for age, parity, tobacco, physical activity, phototype, weekly sun exposure score, energy intake, calcium intake, vitamin D intake, and season. ^e^ Using the geometric mean as the reference. ^f^ Taking into account daily time in sun and skin exposure according to Hanwell et al. ^g^ In tertiles. ^h^ Cut-off point established according to the dietary reference intake for Spanish women aged 40–49 years (*Spanish Federation* of Societies of Nutrition, Food and Dietetics (FESNAD), Ingestas dietéticas de referencia (IDR) para la población española, Eunsa, 2010).

**Table 4:** Association between R1 and R2 metabolite ratios and characteristics of women according to vitamin D status.

	R1: 25(OH)D_3_/Vit D_3_									R2: 1,25(OH)_2_D_3_/25(OH)D_3_								
	25(OH)D_3_ ≤ 50 nmol/L				25(OH)D_3_ > 50 nmol/L					25(OH)D_3_ ≤ 50 nmol/L				25(OH)D_3_ > 50 nmol/L				
Characteristics	GM	GMR ^a^	95%CI	*p*-Val	GM	GMR ^a^	95%CI	*p*-Val	P-Het ^b^	GM	GMR ^c^	95%CI	P-Val	GM	GMR ^c^	95%CI	*p*-Val	P-Het ^b^
Age									0.444									0.698
<45	6.25	1.00			10.27	1.00				3.0 × 10^−3^	1.00			1.7 × 10^−3^	1.00			
≥45	5.88	0.97	(0.89–1.05)	0.397	10.03	1.03	(0.96–1.11)	0.408		3.0 × 10^−3^	1.02	(0.97–1.07)	0.538	1.7 × 10^−3^	0.99	(0.93–1.05)	0.802	
Educational level ^d^									0.962									0.273
Primary school or less	5.99	0.99	(0.87–1.12)	0.840	10.05	0.99	(0.88–1.11)	0.856		3.2 × 10^−3^	1.06	(0.97–1.15)	0.199	1.6 × 10^−3^	0.95	(0.86–1.04)	0.271	
Secondary school	6.06	0.99	(0.92–1.07)	0.878	10.22	0.99	(0.92–1.06)	0.674		3.0 × 10^−3^	0.98	(0.93–1.03)	0.403	1.7 × 10^−3^	1.03	(0.97–1.09)	0.301	
University graduate	6.10	1.02	(0.95–1.10)	0.610	10.12	1.03	(0.96–1.10)	0.459		3.0 × 10^−3^	0.97	(0.92–1.02)	0.183	1.7 × 10^−3^	1.02	(0.97–1.08)	0.421	
Body mass index (kg/m^2^)									0.867									0.565
<20	5.83	1.00			9.28	1.00				3.0 × 10^−3^	1.00			1.7 × 10^−3^	1.00			
20–25	5.91	1.05	(0.92–1.20)	0.453	9.84	1.00	(0.89–1.13)	0.992		3.0 × 10^−3^	1.00	(0.91–1.09)	0.946	1.7 × 10^−3^	0.97	(0.87–1.07)	0.491	
25–29	6.43	1.11	(0.95–1.28)	0.184	10.87	1.10	(0.96–1.26)	0.164		3.0 × 10^−3^	1.01	(0.92–1.11)	0.867	1.6 × 10^−3^	0.92	(0.82–1.03)	0.155	
≥30	6.46	1.14	(0.95–1.36)	0.153	11.92	1.16	(0.97–1.38)	0.098		3.2 × 10^−3^	1.08	(0.96–1.21)	0.179	1.7 × 10^−3^	1.01	(0.87–1.17)	0.895	
Parity									0.113									0.827
Parous	6.06	1.00			10.24	1.00				3.0 × 10^−3^	1.00			1.7 × 10^−3^	1.00			
nuliparous	6.11	1.02	(0.94–1.12)	0.588	9.81	0.93	(0.85–1.02)	0.142		3.1 × 10^−3^	1.06	(1.00–1.12)	0.042	1.7 × 10^−3^	1.05	(0.97–1.13)	0.254	
Tobacco consumption									0.953									0.819
No	6.16	1.00			10.12	1.00				2.9 × 10^−3^	1.00			1.7 × 10^−3^	1.00			
Exsmoker	6.11	0.97	(0.89–1.07)	0.577	9.97	0.99	(0.91–1.07)	0.801		3.0 × 10^−3^	1.04	(0.98–1.10)	0.223	1.7 × 10^−3^	1.01	(0.94–1.08)	0.781	
Current smoker	5.92	0.99	(0.90–1.09)	0.888	10.48	1.02	(0.93–1.12)	0.627		3.1 × 10^−3^	1.04	(0.98–1.11)	0.206	1.7 × 10^−3^	1.01	(0.93–1.09)	0.804	
Alcohol consumption									0.371									0.409
No	6.02	1.00			10.18	1.00				3.0 × 10^−3^	1.00			1.7 × 10^−3^	1.00			
<10 g/day	6.06	1.04	(0.94–1.14)	0.453	10.05	0.96	(0.87–1.06)	0.396		3.0 × 10^−3^	0.99	(0.93–1.05)	0.761	1.7 × 10^−3^	0.93	(0.86–1.00)	0.066	
≥10 g/day	6.14	1.06	(0.93–1.21)	0.379	10.61	1.06	(0.93–1.20)	0.369		2.9 × 10^−3^	0.95	(0.88–1.04)	0.285	1.7 × 10^−3^	0.93	(0.83–1.03)	0.182	
Physical activity (MET-h/week)									0.418									0.959
None	6.05	1.00			10.84	1.00				3.1 × 10^−3^	1.00			1.7 × 10^−3^	1.00			
≤12	6.39	1.02	(0.93–1.13)	0.630	9.89	0.95	(0.86–1.04)	0.236		2.9 × 10^−3^	0.98	(0.92–1.04)	0.448	1.7 × 10^−3^	0.98	(0.91–1.06)	0.687	
>12	5.88	0.93	(0.85–1.03)	0.157	9.68	0.91	(0.83–0.99)	0.031		3.0 × 10^−3^	1.01	(0.95–1.07)	0.860	1.7 × 10^−3^	1.00	(0.93–1.07)	0.932	
Hypercholesterolemia									0.318									0.068
No	6.16	1.00			10.17	1.00				3.0 × 10^−3^	1.00			1.7 × 10^−3^	1.00			
Yes, not treated	5.51	0.93	(0.82–1.05)	0.244	10.05	1.06	(0.94–1.19)	0.374		3.1 × 10^−3^	1.01	(0.94–1.10)	0.737	1.7 × 10^−3^	0.91	(0.82–1.01)	0.064	
Treated with statins	5.68	0.98	(0.76–1.25)	0.846	9.59	0.94	(0.75–1.17)	0.562		3.0 × 10^−3^	0.98	(0.83–1.16)	0.825	1.3 × 10^−3^	0.79	(0.65–0.95)	0.013	
Ever used corticosteroids									0.027									0.545
No	6.16	1.00			10.12	1.00				3.0 × 10^−3^	1.00			1.7 × 10^−3^	1.00			
Yes	5.47	0.86	(0.76–0.98)	0.024	10.45	1.06	(0.95–1.19)	0.271		3.0 × 10^−3^	0.98	(0.90–1.06)	0.636	1.6 × 10^−3^	1.02	(0.93–1.12)	0.682	
Phototype									0.540									0.605
I-II	6.07	1.00			9.95	1.00				3.0 × 10^−3^	1.00			1.7 × 10^−3^	1.00			
III	6.31	1.02	(0.87–1.21)	0.772	10.96	1.15	(0.99–1.33)	0.067		2.9 × 10^−3^	0.96	(0.86–1.07)	0.440	1.6 × 10^−3^	0.91	(0.80–1.03)	0.135	
IV	6.15	1.02	(0.88–1.18)	0.807	10.02	1.06	(0.94–1.20)	0.336		3.0 × 10^−3^	1.00	(0.91–1.10)	0.960	1.7 × 10^−3^	0.97	(0.88–1.08)	0.624	
V-VI	5.77	0.97	(0.82–1.14)	0.673	9.79	1.05	(0.91–1.22)	0.496		3.1 × 10^−3^	1.06	(0.96–1.18)	0.261	1.7 × 10^−3^	0.97	(0.86–1.10)	0.645	
Weekly sun exposure score ^e^									0.023									0.013
<15	5.95	1.00			11.05	1.00				3.2 × 10^−3^	1.00			1.7 × 10^−3^	1.00			
15–28	6.45	1.07	(0.97–1.18)	0.182	10.84	1.09	(0.99–1.20)	0.078		2.8 × 10^−3^	0.94	(0.88–1.00)	0.036	1.6 × 10^−3^	0.98	(0.91–1.07)	0.682	
29–56	5.87	0.99	(0.88–1.13)	0.910	8.30	1.00	(0.89–1.13)	0.951		2.6 × 10^−3^	0.88	(0.81–0.95)	0.001	1.6 × 10^−3^	0.96	(0.87–1.07)	0.482	
Total energy intake (kcal/day) ^f^									0.504									0.625
<1669.2	6.39	1.00			10.14	1.00				3.0 × 10^−3^	1.00			1.6 × 10^−3^	1.00			
1669.2–2144.0	5.91	0.95	(0.86–1.04)	0.270	10.16	0.99	(0.91–1.08)	0.811		2.9 × 10^−3^	1.00	(0.94–1.07)	0.938	1.7 × 10^−3^	1.03	(0.95–1.11)	0.463	
>2144.0	5.90	0.96	(0.87–1.06)	0.435	10.16	1.00	(0.92–1.10)	0.935		3.0 × 10^−3^	1.05	(0.98–1.14)	0.170	1.8 × 10^−3^	1.14	(1.04–1.24)	0.004	
Total calcium intake (mg/day) ^f^									0.234									0.713
<892.5	6.36	1.00			9.85	1.00				3.0 × 10^−3^	1.00			1.7 × 10^−3^	1.00			
892.5–1246.9	5.89	0.93	(0.85–1.03)	0.165	10.03	1.00	(0.92–1.09)	0.988		3.0 × 10^−3^	1.03	(0.96–1.09)	0.405	1.7 × 10^−3^	1.00	(0.93–1.08)	0.968	
>1246.9	5.92	0.97	(0.88–1.07)	0.570	10.58	1.07	(0.97–1.17)	0.156		2.9 × 10^−3^	0.97	(0.91–1.05)	0.475	1.7 × 10^−3^	0.91	(0.83–0.99)	0.033	
Total vitamin D intake (µg/day) ^g^									0.974									0.282
<5	6.04	1.00			10.04	1.00				3.0 × 10^−3^	1.00			1.7 × 10^−3^	1.00			
≥5	5.84	0.98	(0.90–1.08)	0.752	9.77	0.98	(0.89–1.08)	0.666		3.0 × 10^−3^	1.01	(0.95–1.08)	0.742	1.7 × 10^−3^	0.98	(0.91–1.07)	0.719	
Supplements intake	6.95	1.16	(1.00–1.35)	0.047	11.57	1.12	(0.99–1.25)	0.064		2.8 × 10^−3^	0.97	(0.88–1.06)	0.478	1.5 × 10^−3^	0.87	(0.79–0.96)	0.007	
Season ^d^									<0.001									0.020
Spring	5.90	0.98	(0.92–1.05)	0.605	9.58	0.94	(0.89–0.99)	0.032		2.9 × 10^−3^	0.94	(0.90–0.98)	0.004	1.7 × 10^−3^	0.97	(0.92–1.02)	0.193	
Summer	6.12	1.01	(0.92–1.12)	0.781	8.00	0.80	(0.74–0.87)	<0.001		2.6 × 10^−3^	0.94	(0.88–1.01)	0.074	1.6 × 10^−3^	1.00	(0.93–1.07)	0.941	
Fall	6.33	1.01	(0.94–1.08)	0.795	11.91	1.12	(1.05–1.19)	<0.001		3.0 × 10^−3^	1.03	(0.98–1.07)	0.250	1.7 × 10^−3^	0.99	(0.94–1.05)	0.798	
Winter	6.01	0.99	(0.92–1.07)	0.864	12.04	1.19	(1.09–1.29)	<0.001		3.3 × 10^−3^	1.10	(1.05–1.15)	<0.001	1.8 × 10^−3^	1.04	(0.97–1.12)	0.266	

GM: geometric mean; MET: metabolic equivalent.^a^ Geometric mean ratio adjusted for body mass index, parity, physical activity, use of corticosteroids, phototype, weekly sun exposure score, vitamin D intake, and season. ^b^ *p*-value for heterogeneity. ^c^ Geometric mean ratio adjusted for body mass index, parity, phototype, weekly sun exposure score, energy intake, calcium intake, vitamin D intake, and season. ^d^ Using the geometric mean as the reference. ^e^ Taking into account daily time in sun and skin exposure according to Hanwell et al. ^f^ In tertiles. ^g^ Cut-off point established according to the dietary reference intake for Spanish women aged 40–49 years (*Spanish Federation* of Societies of Nutrition, Food and Dietetics (FESNAD), Ingestas dietéticas de referencia (IDR) para la población española, Eunsa, 2010).

**Table 5:** Association between R3 and R4 metabolite ratios and characteristics of women according to vitamin D status.

	R3: 24,25(OH)_2_D_3_/25(OH)D_3_									R4: 24,25(OH)_2_D_3_/1,25(OH)_2_D_3_								
	25(OH)D_3_ ≤ 50 nmol/L				25(OH)D_3_ > 50 nmol/L					25(OH)D_3_ ≤ 50 nmol/L				25(OH)D_3_ > 50 nmol/L				
Characteristics	GM	GMR ^a^	95%CI	*p*-Val	GM	GMR ^a^	95%CI	*p*-Val	P-Het ^b^	GM	GMR ^c^	95%CI	*p*-Val	GM	GMR ^c^	95%CI	*p*-Val	P-Het ^b^
Age									0.543									0.406
<45	0.07	1.00			0.06	1.00				23.48	1.00			36.88	1.00			
≥45	0.07	0.93	(0.88–0.99)	0.020	0.06	0.94	(0.89–1.00)	0.048		21.41	0.92	(0.86–0.98)	0.008	34.95	0.95	(0.89–1.03)	0.197	
Educational level ^d^									0.078									0.455
Primary school or less	0.07	1.08	(0.98–1.19)	0.123	0.06	0.95	(0.87–1.04)	0.266		22.52	1.01	(0.91–1.13)	0.825	34.45	1.00	(0.89–1.13)	0.971	
Secondary school	0.07	0.95	(0.90–1.01)	0.117	0.06	1.05	(0.99–1.11)	0.112		22.06	0.98	(0.91–1.04)	0.486	35.92	1.02	(0.95–1.09)	0.635	
University graduate	0.07	0.97	(0.92–1.03)	0.319	0.06	1.01	(0.95–1.06)	0.819		22.70	1.01	(0.95–1.08)	0.73	36.11	0.98	(0.91–1.05)	0.578	
Body mass index (kg/m^2^)									0.297									0.248
<20	0.06	1.00			0.06	1.00				20.78	1.00			36.01	1.00			
20–25	0.07	1.11	(1.00–1.22)	0.047	0.06	0.98	(0.89–1.08)	0.719		23.38	1.11	(0.99–1.24)	0.066	36.00	1.01	(0.89–1.14)	0.913	
25–29	0.07	1.07	(0.96–1.19)	0.235	0.06	0.99	(0.88–1.10)	0.790		22.15	1.06	(0.94–1.20)	0.321	37.39	1.06	(0.92–1.22)	0.446	
≥30	0.07	1.03	(0.90–1.18)	0.673	0.06	0.97	(0.84–1.12)	0.658		20.46	0.95	(0.81–1.10)	0.461	32.39	0.95	(0.79–1.13)	0.553	
Parity									0.895									0.792
Parous	0.07	1.00			0.06	1.00				23.06	1.00			36.56	1.00			
nuliparous	0.06	0.94	(0.88–1.00)	0.047	0.06	0.93	(0.86–1.00)	0.053		20.98	0.89	(0.83–0.95)	0.001	33.59	0.89	(0.81–0.98)	0.022	
Tobacco consumption									0.994									0.949
No	0.07	1.00			0.06	1.00				23.17	1.00			36.46	1.00			
Exsmoker	0.07	1.02	(0.96–1.09)	0.517	0.06	1.01	(0.94–1.08)	0.845		22.43	0.99	(0.92–1.06)	0.478	36.54	0.99	(0.92–1.08)	0.868	
Current smoker	0.07	0.96	(0.89–1.03)	0.228	0.06	0.96	(0.89–1.03)	0.265		21.64	0.93	(0.86–1.00)	0.064	34.44	0.95	(0.86–1.04)	0.251	
Alcohol consumption									0.161									0.211
No	0.07	1.00			0.06	1.00				23.25	1.00			35.61	1.00			
<10 g/day	0.07	0.97	(0.91–1.05)	0.492	0.06	1.01	(0.93–1.09)	0.898		22.77	1.00	(0.92–1.08)	0.911	35.70	1.09	(0.99–1.20)	0.084	
≥10 g/day	0.06	0.93	(0.85–1.03)	0.171	0.06	1.07	(0.96–1.19)	0.204		21.83	0.98	(0.88–1.09)	0.688	35.91	1.16	(1.01–1.33)	0.032	
Physical activity (MET-h/week)									0.791									0.848
None	0.07	1.00			0.06	1.00				21.85	1.00			34.81	1.00			
≤12	0.07	1.04	(0.96–1.12)	0.334	0.06	1.05	(0.97–1.13)	0.218		22.88	1.08	(1.00–1.17)	0.064	36.71	1.07	(0.97–1.17)	0.168	
>12	0.07	1.03	(0.96–1.10)	0.410	0.06	1.07	(1.00–1.14)	0.067		23.20	1.04	(0.96–1.12)	0.312	36.77	1.06	(0.97–1.16)	0.165	
Hypercholesterolemia									0.621									0.070
No	0.07	1.00			0.06	1.00				22.52	1.00			35.84	1.00			
Yes, not treated	0.07	0.99	(0.91–1.09)	0.913	0.06	0.97	(0.88–1.07)	0.566		21.61	0.99	(0.89–1.09)	0.785	34.68	1.07	(0.94–1.21)	0.303	
Treated with statins	0.07	0.94	(0.78–1.14)	0.547	0.06	1.08	(0.90–1.29)	0.427		22.20	0.95	(0.77–1.17)	0.650	43.67	1.37	(1.09–1.73)	0.006	
Ever used corticosteroids									0.804									0.717
No	0.07	1.00			0.06	1.00				22.52	1.00			35.95	1.00			
Yes	0.07	1.01	(0.92–1.11)	0.828	0.06	1.01	(0.93–1.10)	0.797		22.26	1.03	(0.92–1.14)	0.624	36.15	0.99	(0.88–1.10)	0.809	
Phototype									0.677									0.943
I-II	0.07	1.00			0.06	1.00				22.41	1.00			36.00	1.00			
III	0.07	1.03	(0.91–1.16)	0.637	0.06	0.97	(0.87–1.10)	0.657		23.07	1.07	(0.94–1.23)	0.308	35.08	1.07	(0.92–1.24)	0.399	
IV	0.07	1.03	(0.92–1.15)	0.569	0.06	1.03	(0.93–1.13)	0.618		22.38	1.03	(0.91–1.17)	0.608	35.76	1.05	(0.92–1.19)	0.468	
V-VI	0.07	1.02	(0.90–1.15)	0.765	0.06	0.99	(0.88–1.11)	0.811		22.34	0.97	(0.84–1.11)	0.608	35.84	1.02	(0.88–1.19)	0.794	
Weekly sun exposure score ^e^									0.172									0.508
<15	0.07	1.00			0.06	1.00				21.43	1.00			33.42	1.00			
15–28	0.07	1.03	(0.95–1.10)	0.503	0.06	0.99	(0.92–1.07)	0.879		24.02	1.09	(1.01–1.19)	0.029	36.36	1.02	(0.92–1.12)	0.743	
29–56	0.07	0.98	(0.89–1.08)	0.704	0.06	0.97	(0.88–1.07)	0.510		24.97	1.09	(0.99–1.21)	0.086	38.40	1.02	(0.90–1.15)	0.803	
Total energy intake (kcal/day) ^f^									0.432									0.250
<1669.2	0.07	1.00			0.06	1.00				22.67	1.00			36.98	1.00			
1669.2–2144.0	0.07	0.98	(0.92–1.05)	0.630	0.06	1.00	(0.94–1.08)	0.895		22.38	0.98	(0.90–1.06)	0.606	35.82	0.97	(0.89–1.07)	0.555	
>2144.0	0.07	1.05	(0.97–1.13)	0.211	0.06	1.02	(0.95–1.10)	0.575		23.16	1.01	(0.91–1.11)	0.872	34.28	0.89	(0.80–0.99)	0.033	
Total calcium intake (mg/day) ^f^									0.983									0.879
<892.5	0.07	1.00			0.06	1.00				22.21	1.00			34.35	1.00			
892.5–1246.9	0.07	1.03	(0.96–1.10)	0.466	0.06	1.04	(0.97–1.11)	0.322		22.21	0.98	(0.91–1.07)	0.708	35.88	1.04	(0.95–1.14)	0.378	
>1246.9	0.07	1.04	(0.96–1.12)	0.342	0.06	1.05	(0.97–1.13)	0.215		23.85	1.04	(0.95–1.14)	0.381	36.89	1.18	(1.05–1.31)	0.004	
Total vitamin D intake (µg/day) ^g^									0.783									0.229
<5	0.07	1.00			0.06	1.00				22.50	1.00			35.00	1.00			
≥5	0.07	1.07	(0.99–1.15)	0.069	0.06	1.04	(0.96–1.12)	0.352		23.77	1.04	(0.95–1.13)	0.380	37.21	1.05	(0.95–1.16)	0.364	
Supplements intake	0.06	0.96	(0.86–1.07)	0.466	0.06	0.98	(0.90–1.08)	0.739		22.56	0.98	(0.87–1.11)	0.790	37.98	1.13	(1.00–1.27)	0.042	
Season ^d^									0.002									0.637
Spring	0.06	0.85	(0.81–0.89)	<0.001	0.05	0.88	(0.84–0.92)	<0.001		20.26	0.90	(0.86–0.95)	<0.001	31.54	0.90	(0.85–0.96)	0.001	
Summer	0.07	1.00	(0.92–1.07)	0.914	0.07	1.14	(1.06–1.22)	<0.001		25.08	1.07	(0.98–1.16)	0.134	41.33	1.14	(1.04–1.24)	0.003	
Fall	0.08	1.20	(1.14–1.26)	<0.001	0.07	1.09	(1.04–1.15)	0.001		26.74	1.17	(1.10–1.23)	<0.001	39.88	1.10	(1.04–1.17)	0.002	
Winter	0.07	0.98	(0.93–1.04)	0.509	0.06	0.92	(0.86–0.98)	0.017		20.38	0.89	(0.84–0.95)	<0.001	31.15	0.88	(0.81–0.97)	0.006	

GM: geometric mean; MET: metabolic equivalent. ^a^ Geometric mean ratio adjusted for age, body mass index, parity, tobacco, physical activity, phototype, weekly sun exposure score, vitamin D intake, and season. ^b^ *p*-value for heterogeneity. ^c^ Geometric mean ratio adjusted for age, parity, tobacco, physical activity, phototype, weekly sun exposure score, energy intake, calcium intake, vitamin D intake, and season. ^d^ Using the geometric mean as the reference. ^e^ Taking into account daily time in sun and skin exposure according to Hanwell et al. ^f^ In tertiles. ^g^ Cut-off point established according to the dietary reference intake for Spanish women aged 40–49 years (*Spanish Federation* of Societies of Nutrition, Food and Dietetics (FESNAD), Ingestas dietéticas de referencia (IDR) para la población española, Eunsa, 2010).

## Data Availability

Some or all datasets generated during and/or analyzed during the current study are not publicly available but are available from the corresponding author on reasonable request.

## Supplementary Materials

The following are available online at https://www.mdpi.com/article/10.3390/nu13113747/s1, Table S1: Association between vitamin D metabolite concentrations and characteristics of women. Analysis adjusted for multiple testing. Table S2: Association between vitamin D metabolite ratios and characteristics of women. Analysis adjusted for multiple testing.

## Appendix A

### Appendix A.1. Sample Preparation

A serum aliquot of 240 µL was poured in an amber glass vial and spiked with 10 μL of the deuterated working solution—giving the following final concentrations: 25, 25, 5 and 0.3 ng mL^−1^ of vitamin D_3_-d_3_, 25(OH)D_3_-d_3_, 24,25(OH)_2_D_3_-d_6_ and 1,25(OH)_2_D_3_-d_6_, respectively—, shaken and located into the autosampler. The sample loop was filled with 0.2 mL from the sample vial, which was refrigerated in the autosampler at 6 °C. Shortly, the protocol starts by activation of the SPE sorbent with methanol, followed by a conditioning and equilibration step with 25:75 (*V*/*V)* ACN–water acidified with 0.7% (*V*/*V*) formic acid, the same solution used for sample loading into the cartridge. Under these conditions, the target compounds are retained in the cartridge, which is washed with 30:70 (*V*/*V*) ACN–water to remove retained mid-polar interferents. Then, the chromatographic step starts by switching the left clamp valve of the SPE automated station and putting the cartridge into contact with the initial mobile phase, which also acts as eluent. Elution of the target analytes takes 5 min (longer elution times favor elution of non-polar interferents, which remain retained in the sorbent within the selected interval).

### Appendix A.2. LC–MS/MS Analysis

The initial chromatographic mobile phase was 5 mM ammonium formate in 85:15 (*V*/*V*) methanol–water at a flow rate of 0.5 mL min^−1^. The temperature of the analytical column compartment was set at 15 °C. At min 2, a linear gradient was programmed to obtain a 100% 5 mM ammonium formate in methanol at min 5. The final gradient conditions were maintained for 10 min until the end of the chromatographic separation step. The total analysis time was 15 min, 10 min being required for re-establishing and equilibrating the initial conditions. The chromatographic–detection step of a sample and the SPE step of the next sample overlapped, thus improving the analysis frequency.

The eluate from the chromatographic column was monitored by MS/MS in MRM mode. The flow and temperature of the drying gas (N_2_) were 9 L min^−1^ and 350 °C, respectively. The nebulizer pressure was 50 psi, and the capillary voltage 4750 V in the positive ionization mode.
