# Supplementary material for: Factors Associated with Serum Vitamin D Metabolites and Vitamin D Metabolite Ratios in Premenopausal Women
Source: Nutrients. 2021 Oct 23;13(11):3747. doi: 10.3390/nu13113747 (PMC8621214; doi:10.3390/nu13113747)
Supplement: Supplementary file 1 [file nutrients-13-03747-s001.zip › nutrients-1408740-supplementary.pdf]

Table S1: Association between vitamin D metabolite concentrations and characteristics of women. Analysis adjusted for multiple testing

	Vit D_3				$25(OH)D_3$				$1,25(OH)_2D_3$				$24,25(OH)_2D_3$			
Characteristics	GM	GMR[a]	95%CI	*q*-val[e]	GM	GMR[b]	95%CI	*q*-val[e]	GM	GMR[c]	95%CI	*q*-val[e]	GM	GMR[d]	95%CI	*q*-val[e]
Age																
<45	6.0	1.00			45.9	1.00			108.8	1.00			3.1	1.00		
≥45	6.3	1.02	(0.97-1.06)	0.742	45.9	0.99	(0.95-1.03)	0.832	109.1	1.01	(0.99-1.04)	0.804	2.9	0.93	(0.88-0.98)	0.023
Educational level[f]																
Primary school or less	5.9	0.99	(0.92-1.07)	0.930	43.3	0.97	(0.90-1.04)	0.605	107.6	0.99	(0.95-1.03)	0.916	2.8	0.99	(0.91-1.08)	0.836
Secondary school	6.1	1.01	(0.97-1.06)	0.767	46.9	1.02	(0.98-1.07)	0.506	109.2	1.00	(0.98-1.03)	0.972	3.0	1.02	(0.97-1.07)	0.704
University graduate	6.1	1.00	(0.95-1.04)	0.930	45.6	1.01	(0.97-1.05)	0.833	108.9	1.01	(0.98-1.03)	0.898	3.0	0.99	(0.95-1.04)	0.836
Body mass index (kg/m^2)																
<20	6.5	1.00			45.6	1.00			107.5	1.00			2.8	1.00		
20-25	6.3	0.98	(0.90-1.06)	0.755	46.5	1.00	(0.93-1.08)	0.949	109.2	1.00	(0.95-1.04)	0.983	3.1	1.04	(0.95-1.14)	0.610
25-29	5.8	0.92	(0.85-1.01)	0.284	45.5	0.97	(0.89-1.05)	0.646	108.3	0.98	(0.93-1.03)	0.804	2.9	0.99	(0.90-1.09)	0.836
≥30	5.3	0.83	(0.74-0.93)	0.008	43.6	0.90	(0.81-1.00)	0.180	110.4	1.00	(0.94-1.06)	0.983	2.7	0.90	(0.80-1.01)	0.197
Parity																
Parous	6.1	1.00			46.7	1.00			108.6	1.00			3.1	1.00		
nuliparous	6.1	0.98	(0.93-1.04)	0.725	43.4	0.90	(0.86-0.95)	0.004	110.1	1.02	(0.99-1.05)	0.617	2.7	0.87	(0.81-0.92)	0.003
Tobacco consumption																
No	6.2	1.00			46.6	1.00			108.5	1.00			3.0	1.00		
Exsmoker	6.2	1.01	(0.96-1.07)	0.790	46.2	1.00	(0.95-1.05)	0.949	109.5	1.01	(0.98-1.04)	0.804	3.0	1.02	(0.96-1.08)	0.754
Current smoker	6.0	0.99	(0.94-1.05)	0.930	44.5	0.96	(0.91-1.01)	0.384	108.8	1.02	(0.98-1.05)	0.804	2.8	0.93	(0.87-0.99)	0.053
Alcohol consumption																
No	6.0	1.00			43.9	1.00			109.1	1.00			3.0	1.00		
< 10 g/day	6.2	1.00	(0.94-1.06)	0.973	46.2	1.04	(0.98-1.10)	0.396	107.8	0.98	(0.95-1.02)	0.804	3.0	1.01	(0.94-1.08)	0.836
≥10 g/day	6.1	0.98	(0.90-1.06)	0.742	46.8	1.07	(0.99-1.15)	0.256	110.0	1.00	(0.96-1.05)	0.983	3.0	1.04	(0.95-1.13)	0.641
Physical activity (MET-h/week)																
None	5.9	1.00			44.2	1.00			108.9	1.00			2.8	1.00		
≤12	6.1	1.02	(0.96-1.08)	0.725	45.8	1.04	(0.99-1.10)	0.384	108.2	1.00	(0.97-1.03)	0.983	3.0	1.08	(1.01-1.15)	0.053
>12	6.5	1.08	(1.02-1.14)	0.038	48.2	1.05	(1.00-1.11)	0.238	109.4	1.01	(0.98-1.05)	0.804	3.2	1.08	(1.01-1.15)	0.053
Hypercholesterolemia																
No	6.1	1.00			45.9	1.00			109.0	1.00			3.0	1.00		
Yes, not treated	6.5	1.05	(0.98-1.13)	0.474	45.1	1.02	(0.95-1.10)	0.752	110.3	0.99	(0.95-1.04)	0.972	2.9	1.01	(0.93-1.10)	0.836
Treated with statins	7.0	1.08	(0.93-1.26)	0.596	51.6	1.04	(0.91-1.20)	0.746	103.3	0.93	(0.85-1.01)	0.416	3.2	1.03	(0.88-1.22)	0.836
Use of corticosteroids																
No	6.1	1.00			45.7	1.00			108.8	1.00			3.0	1.00		
Yes	6.6	1.09	(1.01-1.17)	0.105	47.7	1.05	(0.98-1.12)	0.416	110.5	1.02	(0.98-1.06)	0.804	3.0	1.05	(0.96-1.13)	0.516
Phototype																
I-II	6.3	1.00			47.4	1.00			111.8	1.00			3.1	1.00		

III	5.9	0.93	(0.85-1.03)	0.474	45.7	0.96	(0.87-1.04)	0.551	107.6	0.94	(0.89-0.99)	0.269	2.9	0.97	(0.87-1.08)	0.754
IV	6.1	0.95	(0.87-1.03)	0.474	46.4	0.97	(0.90-1.05)	0.736	108.8	0.97	(0.92-1.01)	0.692	3.0	1.00	(0.91-1.10)	0.980
V-VI	6.3	0.97	(0.88-1.06)	0.725	43.8	0.90	(0.83-0.99)	0.119	110.1	0.98	(0.93-1.04)	0.819	2.9	0.92	(0.83-1.03)	0.292
Weekly sun exposure score[g]																
<15	5.9	1.00			41.9	1.00			110.7	1.00			2.7	1.00		
15-28	6.0	0.96	(0.91-1.02)	0.474	49.8	1.13	(1.07-1.20)	0.004	107.2	0.98	(0.94-1.03)	0.804	3.2	1.11	(1.05-1.19)	0.005
29-56	7.4	1.08	(1.00-1.16)	0.176	53.1	1.16	(1.08-1.24)	0.004	105.8	0.95	(0.87-1.04)	0.804	3.4	1.11	(1.03-1.21)	0.040
Total energy intake (kcal/day)[h]																
<1669.2	6.0	1.00			46.1	1.00			106.7	1.00			3.0	1.00		
1669.2 - 2144.0	6.3	1.04	(0.98-1.10)	0.474	46.6	1.00	(0.95-1.05)	0.949	107.8	1.01	(0.97-1.04)	0.931	2.9	0.97	(0.91-1.04)	0.641
>2144.0	6.2	1.00	(0.94-1.06)	0.973	44.8	0.97	(0.91-1.02)	0.456	110.6	1.04	(1.00-1.07)	0.269	3.0	0.97	(0.90-1.04)	0.619
Total calcium intake (mg/day)[h]																
<892.5	6.0	1.00			45.2	1.00			108.4	1.00			2.9	1.00		
892.5 - 1246.9	6.2	1.03	(0.97-1.09)	0.679	46.1	1.01	(0.96-1.07)	0.843	109.3	1.00	(0.97-1.03)	0.983	3.0	1.03	(0.97-1.10)	0.610
>1246.9	6.2	0.99	(0.94-1.06)	0.930	46.2	1.02	(0.97-1.08)	0.674	107.5	0.96	(0.92-1.00)	0.269	3.1	1.06	(0.99-1.13)	0.220
Total vitamin D intake (μg/day)[i]																
<5	6.1	1.00			45.1	1.00			108.0	1.00			2.9	1.00		
≥5	6.4	1.04	(0.98-1.10)	0.474	45.7	1.01	(0.95-1.06)	0.890	110.0	1.01	(0.98-1.05)	0.819	3.1	1.06	(0.99-1.13)	0.220
Supplements intake	5.8	0.96	(0.88-1.04)	0.597	52.8	1.18	(1.09-1.27)	0.004	107.7	1.00	(0.96-1.05)	0.994	3.2	1.12	(1.02-1.22)	0.053
Season[f]																
Spring	6.4	1.05	(1.01-1.09)	0.069	46.2	1.02	(0.99-1.06)	0.416	107.0	0.97	(0.95-0.99)	0.048	2.6	0.88	(0.84-0.91)	0.003
Summer	7.5	1.13	(1.06-1.19)	0.008	53.6	1.06	(1.01-1.12)	0.112	107.3	0.99	(0.96-1.02)	0.804	3.6	1.12	(1.05-1.19)	0.003
Fall	5.5	0.92	(0.89-0.96)	0.008	46.0	0.99	(0.96-1.03)	0.890	108.2	1.00	(0.97-1.02)	0.931	3.4	1.14	(1.09-1.19)	0.003
Winter	5.6	0.92	(0.88-0.96)	0.008	40.6	0.92	(0.88-0.97)	0.006	114.1	1.05	(1.02-1.08)	0.032	2.6	0.89	(0.85-0.94)	0.003

GM: geometric mean; MET: metabolic equivalent.
[a] Geometric mean ratio adjusted for body mass index, physical activity, use of corticosteroids, weekly sun exposure score, vitamin D intake, and season.
[b] Geometric mean ratio adjusted for body mass index, parity, physical activity, weekly sun exposure score, vitamin D intake, and season.
[c] Geometric mean ratio adjusted for parity, energy intake, weekly sun exposure score, vitamin D intake, and season.
[d] Geometric mean ratio adjusted for age, body mass index, parity, tobacco, physical activity, weekly sun exposure score, calcium intake, vitamin D intake, and season.
[e] p-value following the Benjamini and Hochberg procedure.
[f] Using the geometric mean as the reference.
[g] Taking into account daily time in sun and skin exposure according to Hanwell et al.
[h] In tertiles.
[i] Cut-off point established according to the dietary reference intake for Spanish women aged 40–49 years (*Spanish Federation* of Societies of Nutrition, Food and Dietetics (FESNAD), Ingestas dietéticas de referencia (IDR) para la población española, Eunsa, 2010).

Table S2: Association between vitamin D metabolite ratios and characteristics of women. Analysis adjusted for multiple testing

	R1: 25(OH)D_3 /Vit D_3				R2: 1,25$(OH)_2D_3$ /25(OH)D_3				R3: 24,25$(OH)_2D_3$/25(OH)D_3				R4: 24,25$(OH)_2D_3$/1,25$(OH)_2D_3$			
Characteristics	GM	GMR[a]	95%CI	*q*-val[e]	GM	GMR[b]	95%CI	*q*-val[e]	GM	GMR[c]	95%CI	*q*-val[e]	GM	GMR[d]	95%CI	*q*-val[e]
Age																
<45	7.66	1.00			$2.4×10^{-3}$	1.00			0.07	1.00			28.24	1.00		
>45	7.31	0.98	(0.92-1.04)	0.794	$2.4×10^{-3}$	1.02	(0.97-1.07)	0.839	0.06	0.94	(0.90-0.98)	0.043	26.13	0.92	(0.88-0.97)	0.012
Educational level[f]																
Primary school or less	7.29	0.99	(0.89-1.10)	0.875	$2.5×10^{-3}$	1.00	(0.92-1.08)	0.978	0.07	1.02	(0.95-1.09)	0.741	26.48	1.01	(0.93-1.10)	0.838
Secondary school	7.65	1.00	(0.94-1.07)	0.908	$2.3×10^{-3}$	0.99	(0.94-1.04)	0.852	0.06	0.99	(0.95-1.03)	0.741	27.42	1.00	(0.95-1.06)	0.892
University graduate	7.43	1.01	(0.95-1.07)	0.875	$2.4×10^{-3}$	1.01	(0.96-1.06)	0.852	0.06	0.99	(0.95-1.03)	0.741	27.19	0.98	(0.93-1.04)	0.644
Body mass index (kg/m^2)																
<20	7.04	1.00			$2.4×10^{-3}$	1.00			0.06	1.00			25.97	1.00		
20-25	7.35	1.04	(0.93-1.16)	0.794	$2.3×10^{-3}$	0.99	(0.91-1.08)	0.902	0.07	1.05	(0.97-1.12)	0.514	28.13	1.06	(0.97-1.16)	0.320
25-29	7.80	1.06	(0.94-1.20)	0.794	$2.4×10^{-3}$	1.03	(0.94-1.13)	0.852	0.06	1.04	(0.96-1.13)	0.636	26.86	1.02	(0.92-1.13)	0.749
>30	8.15	1.09	(0.94-1.27)	0.794	$2.5×10^{-3}$	1.11	(0.99-1.25)	0.195	0.06	1.01	(0.91-1.11)	0.916	24.35	0.91	(0.80-1.03)	0.284
Parity																
Parous	7.60	1.00			$2.3×10^{-3}$	1.00			0.07	1.00			28.13	1.00		
nuliparous	7.16	0.92	(0.85-0.99)	0.240	$2.5×10^{-3}$	1.13	(1.06-1.19)	0.003	0.06	0.95	(0.91-1.00)	0.245	24.54	0.84	(0.79-0.90)	0.004
Tobacco consumption																
No	7.56	1.00			$2.3×10^{-3}$	1.00			0.07	1.00			27.94	1.00		
Exsmoker	7.50	1.00	(0.93-1.07)	0.908	$2.4×10^{-3}$	1.01	(0.95-1.07)	0.852	0.07	1.01	(0.97-1.06)	0.741	27.53	1.00	(0.94-1.06)	0.974
Current smoker	7.38	0.99	(0.91-1.07)	0.875	$2.4×10^{-3}$	1.04	(0.98-1.11)	0.392	0.06	0.96	(0.91-1.02)	0.514	25.88	0.93	(0.87-0.99)	0.075
Alcohol consumption																
No	7.26	1.00			$2.5×10^{-3}$	1.00			0.07	1.00			27.08	1.00		
< 10 g/day	7.50	1.05	(0.97-1.14)	0.794	$2.3×10^{-3}$	0.93	(0.88-0.99)	0.083	0.06	0.97	(0.92-1.03)	0.602	27.52	1.05	(0.98-1.13)	0.268
>10 g/day	7.70	1.10	(0.99-1.23)	0.416	$2.4×10^{-3}$	0.90	(0.83-0.98)	0.064	0.06	0.97	(0.90-1.04)	0.636	26.84	1.08	(0.98-1.18)	0.256
Physical activity (MET-h/week)																
None	7.49	1.00			$2.5×10^{-3}$	1.00			0.06	1.00			25.92	1.00		
<12	7.58	1.03	(0.95-1.11)	0.794	$2.4×10^{-3}$	0.95	(0.90-1.01)	0.284	0.06	1.03	(0.98-1.09)	0.514	27.52	1.10	(1.03-1.17)	0.019
>12	7.44	0.97	(0.90-1.05)	0.794	$2.3×10^{-3}$	0.96	(0.90-1.02)	0.312	0.07	1.03	(0.98-1.08)	0.514	28.83	1.09	(1.02-1.16)	0.023
Hypercholesterolemia																
No	7.56	1.00			$2.0×10^{-3}$	1.00			0.06	1.00			27.22	1.00		
Yes, not treated	6.96	0.96	(0.86-1.06)	0.794	$2.4×10^{-3}$	0.98	(0.90-1.06)	0.852	0.06	0.99	(0.93-1.06)	0.903	25.96	1.02	(0.93-1.11)	0.781
Treated with statins	7.38	0.97	(0.80-1.19)	0.875	$2.0×10^{-3}$	0.88	(0.75-1.03)	0.284	0.06	0.99	(0.87-1.14)	0.916	31.14	1.12	(0.94-1.33)	0.320

Use of corticosteroids																
No	7.53	1.00			2.4×10^{-3}	1.00			0.06	1.00			27.24	1.00		
Yes	7.21	0.96	(0.87-1.06)	0.794	2.3×10^{-3}	0.98	(0.91-1.06)	0.852	0.06	1.01	(0.94-1.08)	0.903	27.36	1.02	(0.94-1.11)	0.723
Phototype																
I-II	7.46	1.00			2.4×10^{-3}	1.00			0.06	1.00			27.33	1.00		
III	7.73	1.02	(0.89-1.16)	0.875	2.4×10^{-3}	0.99	(0.89-1.09)	0.882	0.06	1.02	(0.94-1.11)	0.781	26.93	1.04	(0.93-1.16)	0.644
IV	7.60	1.02	(0.91-1.15)	0.875	2.3×10^{-3}	1.00	(0.91-1.09)	0.978	0.06	1.03	(0.96-1.11)	0.686	27.43	1.04	(0.94-1.15)	0.573
V-VI	6.99	0.93	(0.82-1.06.)	0.794	2.5×10^{-3}	1.09	(0.98-1.20)	0.283	0.07	1.02	(0.94-1.12)	0.741	26.50	0.95	(0.85-1.06)	0.554
Weekly sun exposure score[g]																
<15	7.14	1.00			2.6×10^{-3}	1.00			0.06	1.00			24.42	1.00		
15-28	8.33	1.16	(1.08-1.26)	0.016	2.2×10^{-3}	0.88	(0.83-0.93)	0.003	0.06	0.98	(0.93-1.04)	0.741	29.46	1.12	(1.04-1.19)	0.005
29-56	7.19	1.08	(0.97-1.19)	0.699	2.0×10^{-3}	0.82	(0.76-0.89)	0.003	0.06	0.96	(0.90-1.03)	0.514	32.16	1.15	(1.05-1.25)	0.009
Total energy intake (kcal/day)[h]																
<1669.2	7.75	1.00			2.3×10^{-3}	1.00			0.06	1.00			27.82	1.00		
1669.2 - 2144.0	7.41	0.96	(0.89-1.04)	0.794	2.3×10^{-3}	1.04	(0.97-1.10)	0.459	0.06	0.99	(0.95-1.05)	0.903	27.24	0.96	(0.89-1.02)	0.320
>2144.0	7.28	0.97	(0.89-1.05)	0.794	2.5×10^{-3}	1.13	(1.05-1.22)	0.005	0.07	1.04	(0.98-1.09)	0.514	26.95	0.93	(0.86-1.00)	0.151
Total calcium intake (mg/day)[h]																
<892.5	7.54	1.00			2.4×10^{-3}	1.00			0.06	1.00			26.30	1.00		
892.5 - 1246.9	7.39	0.98	(0.91-1.06)	0.875	2.4×10^{-3}	0.99	(0.93-1.05)	0.855	0.06	1.02	(0.97-1.08)	0.675	27.26	1.03	(0.96-1.10)	0.595
>1246.9	7.50	1.03	(0.95-1.12)	0.794	2.3×10^{-3}	0.91	(0.85-0.98)	0.060	0.07	1.03	(0.98-1.09)	0.514	28.49	1.12	(1.03-1.21)	0.018
Total vitamin D intake (µg/day)[i]																
<5	7.39	1.00			2.4×10^{-3}	1.00			0.06	1.00			26.81	1.00		
≥5	7.13	0.97	(0.89-1.05)	0.794	2.4×10^{-3}	1.01	(0.95-1.08)	0.852	0.07	1.06	(1.01-1.12)	0.184	28.28	1.03	(0.96-1.11)	0.554
Supplements intake	9.11	1.21	(1.08-1.35)	0.016	2.0×10^{-3}	0.86	(0.79-0.93)	0.003	0.06	0.96	(0.89-1.03)	0.514	29.77	1.10	(1.01-1.21)	0.094
Season[f]																
Spring	7.17	0.98	(0.94-1.04)	0.794	2.3×10^{-3}	0.94	(0.91-0.98)	0.014	0.06	0.86	(0.83-0.88)	0.008	24.22	0.91	(0.87-0.95)	0.004
Summer	7.15	0.93	(0.86-1.01)	0.416	2.0×10^{-3}	0.95	(0.90-1.01)	0.284	0.07	1.05	(1.00-1.11)	0.245	33.53	1.12	(1.05-1.20)	0.005
Fall	8.29	1.08	(1.02-1.14)	0.053	2.4×10^{-3}	0.99	(0.95-1.03)	0.852	0.07	1.14	(1.10-1.19)	0.008	31.71	1.15	(1.10-1.20)	0.004
Winter	7.23	1.01	(0.94-1.08)	0.875	2.8×10^{-3}	1.12	(1.06-1.18)	0.003	0.06	0.97	(0.93-1.01)	0.514	22.81	0.86	(0.81-0.91)	0.004

GM: geometric mean; MET: metabolic equivalent.

[a] Geometric mean ratio adjusted for body mass index, parity, physical activity, use of corticosteroids, phototype, weekly sun exposure score, vitamin D intake, and season.

[b] Geometric mean ratio adjusted for body mass index, parity, phototype, weekly sun exposure score, energy intake, calcium intake, vitamin D intake, and season.

[c] Geometric mean ratio adjusted for age, body mass index, parity, tobacco, physical activity, phototype, weekly sun exposure score, vitamin D intake, and season.

[d] Geometric mean ratio adjusted for age, parity, tobacco, physical activity, phototype, weekly sun exposure score, energy intake, calcium intake, vitamin D intake, and season.

[e] p-value following the Benjamini and Hochberg procedure.

[f] Using the geometric mean as the reference.

[g] Taking into account daily time in sun and skin exposure according to Hanwell et al.

[h] In tertiles.

[i] Cut-off point established according to the dietary reference intake for Spanish women aged 40–49 years (*Spanish Federation* of Societies of Nutrition, Food and Dietetics (FESNAD), Ingestas dietéticas de referencia (IDR) para la población española, Eunsa, 2010).
